# Genetic variants in *DBC1*, *SIRT1*, *UCP2* and *ADRB2* as potential biomarkers for severe obesity and metabolic complications

**DOI:** 10.3389/fgene.2024.1363417

**Published:** 2024-05-22

**Authors:** Ana Carolina Proença da Fonseca, Izadora Sthephanie da Silva Assis, Kaio Cezar Rodrigues Salum, Lohanna Palhinha, Gabriella de Medeiros Abreu, Verônica Marques Zembrzuski, Mario Campos Junior, José Firmino Nogueira-Neto, Amanda Cambraia, Mauro Lucio Ferreira Souza Junior, Clarissa Menezes Maya-Monteiro, Pedro Hernán Cabello, Patrícia Torres Bozza, João Regis Ivar Carneiro

**Affiliations:** ^1^ Laboratory of Immunopharmacology, Oswaldo Cruz Institute, Oswaldo Cruz Foundation, Rio de Janeiro, Brazil; ^2^ Human Genetics Laboratory, Oswaldo Cruz Institute, Oswaldo Cruz Foundation, Rio de Janeiro, Brazil; ^3^ Genetics Laboratory, Grande Rio University/AFYA, Rio de Janeiro, Brazil; ^4^ Postgraduate Program in Translational Biomedicine, Grande Rio University/AFYA, Rio de Janeiro, Brazil; ^5^ Clementino Fraga Filho University Hospital, Federal University of Rio de Janeiro, Rio de Janeiro, Brazil; ^6^ Josué de Castro Nutrition Institute, Federal University of Rio de Janeiro, Rio de Janeiro, Brazil; ^7^ Department of Pathology and Laboratory, Rio de Janeiro State University, Rio de Janeiro, Brazil; ^8^ Laboratory of Human and Medical Genetics, Institute of Biological Sciences, Federal University of Pará, Belém, Brazil

**Keywords:** DBC1, SIRT1, biomarkers, severe obesity, metabolic complications

## Abstract

**Introduction:**

Obesity is a multifactorial disease associated with the development of many comorbidities. This disease is associated with several metabolic alterations; however, it has been shown that some individuals with obesity do not exhibit metabolic syndrome. Adipose tissue neutralizes the detrimental effects of circulating fatty acids, ectopic deposition, and inflammation, among others, through its esterification into neutral lipids that are stored in the adipocyte. However, when the adipocyte is overloaded, i.e., its expansion capacity is exceeded, this protection is lost, resulting in fatty acid toxicity with ectopic fat accumulation in peripheral tissues and inflammation. In this line, this study aimed to investigate whether polymorphisms in genes that control adipose tissue fat storage capacity are potential biomarkers for severe obesity susceptibility and also metabolic complications.

**Methods:**

This study enrolled 305 individuals with severe obesity (cases, BMI≥35 kg/m^2^) and 196 individuals with normal weight (controls, 18.5≤BMI≤24.9 kg/m^2^). Demographic, anthropometric, biochemical, and blood pressure variables were collected from the participants. Plasma levels of leptin, resistin, MCP1, and PAI1 were measured by Bio-Plex 200 Multiplexing Analyzer System. Genomic DNA was extracted and variants in *DBC1* (rs17060940), *SIRT1* (rs7895833 and rs1467568), *UCP2* (rs660339), *PPARG* (rs1801282) and *ADRB2* (rs1042713 and rs1042714) genes were genotyped by PCR allelic discrimination using TaqMan^®^ assays.

**Results:**

Our findings indicated that *SIRT1* rs7895833 polymorphism was a risk factor for severe obesity development in the overdominant model. *SIRT1* rs1467568 and *UCP2* rs660339 were associated with anthropometric traits. *SIRT1* rs1467568 G allele was related to lower medians of body adipose index and hip circumference, while the *UCP2* rs660339 AA genotype was associate with increased body mass index. Additionally, *DBC1* rs17060940 influenced glycated hemoglobin. Regarding metabolic alterations, 27% of individuals with obesity presented balanced metabolic status in our cohort. Furthermore, *SIRT1* rs1467568 AG genotype increased 2.5 times the risk of developing metabolic alterations. No statistically significant results were observed with *Peroxisome Proliferator-Activated Receptor Gama* and *ADRB2* polymorphisms.

**Discussion/Conclusion:**

This study revealed that *SIRT1* rs7895833 and rs1467568 are potential biomarkers for severe obesity susceptibility and the development of unbalanced metabolic status in obesity, respectively. *UCP2* rs660339 and *DBC1* rs17060940 also showed a significant role in obesity related-traits.

## Introduction

Obesity continues to grow as a serious public health concern. Based on the latest data from the NCD Risk Factor Collaboration, almost 650 million adults had obesity in 2016 (defined by a body mass index [BMI] of ≥30 kg/m^2^) (WHO, 2022). The situation is just as alarming since it is estimated that 1 billion adults will exhibit this phenotype in 2025 (Abarca-Gómez et al., 2017). Obesity is associated with reduced life expectancy due to increased mortality from chronic diseases, such as type 2 diabetes mellitus (T2DM), hypertension, cardiovascular disease, and cancers (WHO, 2022). More recently, it has been highlighted that individuals with obesity present an increased risk for severity and mortality in COVID-19 and Influenza infections (Zhao et al., 2020).

Although obesity is commonly seen as the primary cause of metabolic syndrome and other human metabolic diseases, there is a large variation in the subject’s risk of developing these comorbidities. The effective fat storage in the adipose tissue is suggested to be a key factor in the protection against metabolic dysfunctions. When the capacity of fat storage in adipose tissue depots is exceeded, the excess fat can accumulate in peripheral tissues and cause inflammatory and metabolic dysfunctions in a process known as lipotoxicity (Listenberger et al., 2003; Wang et al., 2008; Unger and Scherer, 2010; Almandoz et al., 2013; Escande et al., 2015). In this context, several studies focus on the molecular pathways involved in adipose tissue fat storage capacity and inflammation, since adipocyte fat accumulation would delay the development of metabolic disease (Lê et al., 2011; Escande et al., 2015; Moreno-Navarrete et al., 2015).

*DBC1* gene (*Deleted in breast cancer 1*) encodes a pleiotropic nuclear protein which induces the expression of nuclear factor kappa B (NF-kB) that regulates inflammatory cytokines through inhibition of sirtuin 1 activity (Lê et al., 2011; Escande et al., 2015; Moreno-Navarrete et al., 2015). Moreno-Navarrete and coworkers (2015) reported that *DBC1* knockdown (KD) in mice resulted in a significant reduction in the expression of inflammatory genes and increased intracellular lipid accumulation. These results suggest that the DBC1 KD mice are protected against adipose tissue inflammation (Moreno-Navarrete et al., 2015). Escande and colleagues (2015) also observed that the DBC1 knockout increased the fat accumulation capacity *in vitro* and *in vivo*, resulting in a “healthy obesity” phenotype (Escande et al., 2015). In this context, the increased activation of NF-kB in adipose tissue is associated with decreased adipogenic capacity (Lê et al., 2011).

Sirtuin 1, encoded by *SIRT1* gene, is a nicotinamide adenine dinucleotide (NAD+)-dependent protein deacetylase that has anti-inflammatory activities in adipose tissue by direct deacetylation of NF-kB and chromatin remodeling at inflammatory gene promoters (Kotas et al., 2013; Moreno-Navarrete et al., 2015). SIRT1 also acts as a cellular energy sensor and drives glucose and fat metabolism by regulating the expression of important genes, such as *PPARG* and *UCP2* (Picard et al., 2004; Bordone et al., 2006; Chaudhary and Pfluger, 2009). Picard and coworkers (2014) showed that SIRT is a suppressor of adipogenesis by inhibiting the expression of genes responsible for promoting the differentiation of white adipocytes and lipid accumulation (Picard et al., 2004).

*PPARG* (Peroxisome Proliferator-Activated Receptor Gama) gene encodes two major isoforms (PPARγ1 and PPARγ2). PPARγ2 is highly expressed in adipose tissue and, upon activation, forms a heterodimer with retinoid X receptor, regulating the expression of numerous target genes (Spiegelman, 1998; Auwerx, 1999). PPARG plays an important role in adipose tissue formation and also regulates lipid and glucose metabolism. Therefore, PPARG is a known target in the study of T2DM and obesity (Luan et al., 2001; Ali et al., 2009; Hasan et al., 2017; Bakhashab et al., 2020). PPARG is repressed by SIRT1, which results in attenuated adipogenesis and increased lipolysis in white adipose tissue (Picard et al., 2004; Chaudhary and Pfluger, 2009; Dmitrenko et al., 2022). In addition, the reduction of PPARG also results in the improvement of insulin resistance and inhibition of glucose production (Spiegelman, 1998; Auwerx, 1999; Bakhashab et al., 2020).

*UCP2* encodes an anion-carrier protein, called uncoupling protein 2. This protein is located in the mitochondrial inner membrane which participates in the energy metabolism of cells (Nicoletti et al., 2017). In humans, five UCP members were identified (UCP1—UCP5). UCP2 is expressed in a wide range of tissues, including the pancreas, central nervous system, and white adipose tissue. UCP2 plays an important role in inhibiting the formation of reactive oxygen species, negative regulation of insulin secretion by pancreatic beta cells, and fatty acid metabolism, which are mechanisms associated with metabolic disease (Pecqueur et al., 2001; Azzu and Brand, 2010; Souza et al., 2011). Interestingly, PPARγ increases the expression of *UCP2*, while SIRT1 inhibits the expression (Affourtit and Brand, 2008). During food deprivation, Sirt1 negatively regulates UCP2, leading to ATP production and increasing insulin secretion by pancreatic β cells (Chaudhary and Pfluger, 2009).

Lastly, the β2-adrenergic receptor is a major lipolytic receptor in human adipocytes, codified by the *ADRB2* gene. This receptor stimulated by catecholamines in adipose tissue, increases lipolysis and resultsin the reduction of adipocyte size and lipid content (Lafontan et al., 2000; Jalba et al., 2008; Kurylowicz et al., 2015). The expression of *ADRB2* is lower in adipose tissue of subjects with obesity when compared to normal-weight individuals. Furthermore, lower expression levels of *ADRB2* were observed in visceral adipose tissue when compared to subcutaneous adipose tissue of subjects with obesity (Kurylowicz et al., 2015).

Obesity and comorbidities have a significant genetic component, and the disease development is dependent on the interaction between genetic background and environmental factors (da Fonseca et al., 2017). However, there are still controversies and gaps about the responsible genes, their common variants, and obesity (or metabolic complications in obesity). In the present study, we have investigated common variants in *DBC1* (rs17060940), *SIRT1* (rs7895833 and rs1467568), *UCP2* (rs660339), *PPARG* (rs1801282) and *ADRB2* (rs1042713 and rs1042714) (Table 1).

**TABLE 1:** Previous findings of the candidate genes and common variants selected in the study.

Gene	Chromosome location	Biological role	dbSNP	SNP location	MAF	Previous studies	References
*DBC1*	8p21.3	Pleiotropic nuclear protein which acts as an inhibitor of histone deacetylase, plays a crucial role as a modulator of epigenetics and metabolic function	rs17060940	Splice polypyrimidine tract variant	0.14	None	
*SIRT1*	10q21.3	Nicotinamide adenine dinucleotide (NAD+)-dependent histone deacetylase which controls the expression of obesity genes related to adipogenesis	rs7895833	Intergenic variant	0.39	- Associated with BMI, WC, and obesity susceptibility in Korean children	Lee et al., 2016
						- Associated with obesity in Turkey, Dutch, and Japanese studies; however, the mutant allele (G) was a risk factor only in the Turkey population	Kilic et al. (2015), Zillikens et al. (2009), Shimoyama et al. (2011)
						- The wild type allele (A) was a risk factor for overweight in women who did not control their caloric intake. This association was not observed in women who restricted calories or practiced physical exercises	Higashibata et al. (2016)
						- No relation with overweight/obesity susceptibility in a Brazilian adultadult cohort	Meneguette et al. (2016)
						- Risk factor for metabolic syndrome in a Chinese Han population, but not in a Brazilian cohort	Meneguette et al. (2016), Tao et al. (2022)
			rs1467568	Intronic variant	0.32	- Protective factor for obesity in a Dutch and French population	Clark et al. (2012), Zillikens et al. (2009)
						- Associated with weight loss resistance in a therapy treatment in a sample from Spain	Garaulet et al. (2012)
						- No relationship with early-onset coronary artery disease in South African Indians	Ramkaran et al., 2016
*PPARG*	3p25	Encodes a nuclear receptor that acts in adipogenesis as well as lipid and glucose metabolism	rs1801282	Missense variant	0.07	- No association with BMI, HbA1c, FBS, triglyceride and cholesterol in T2DM individuals from Saudi population	Bakhashab et al. (2020)
						- Associated with obesity susceptibility in male individuals from Tunisian and in females from Taiwan	Ali et al. (2009), Hsiao and Lin (2015)
						- Positively associated with anthropometric parameters (BMI, body weight, and/or WC) and obesity susceptibility in Caucasian and North Indian subjects	Beamer et al. (1998), Prakash et al. (2012)
						- Mutant allele (G) associated with higher BMI, fat mass, WC, and also increased visceral and subcutaneous adipose tissue area in Québec families	Robitaille et al., 2003
*UCP2*	11q13.4	Mitochondrial uncoupling protein 2 which has a key role in energy metabolism of cells	rs660339	Missense variant p. (Ala55Val)	0.42	- Homozygote AA was a risk factor for obesity and T2DM in Chinese and North American subjects	Xiu et al. (2004), Yu et al. (2005)
						- No relation with obesity, BMI, and/or T2DM in Japanese and Indonesian cohorts	Kubota et al. (1998), Saraswati et al. (2011)
						- No linked to obesity risk in Asian or of European descent (meta- analysis study)	Qian et al. (2013)
						- Associated with weight loss 1 year after RYGB, being suggested as a biomarker	Nicoletti et al. (2017)
*ADRB2*	5q31–32	β2- adrenergic receptor which plays a significant role in the metabolism of lipids and thermogenesis	rs1042714	Missense variant p. (Gln27Glu)	0.20	- Linked to obesity risk in the meta-analysis study conducted with 9,995 subjects	Zhang et al. (2014)
						- No relation with obesity in cohorts from Germany, Italy, and Australia	Kortner et al. (1999), Galletti et al. (2004), Oberkofler et al. (2000)
						- A risk factor for obesity in Asians, Pacific Islanders, and American Indians, but not in Europeans (meta-analysis study)	Jalba et al. (2008)
						- Associated with a better cardiometabolic response after 12 weeks of physical exercise program (3 sessions per week)	Silva et al., 2022

In the literature, it was observed a significant increase in the *SIRT1* protein level with age, and found that older individuals carrying rs7895833 AG genotype had the highest *SIRT1* levels. This suggests that the rs7895833 polymorphism may be associated with differences in *SIRT1* protein expression, potentially through mechanisms that affect gene expression or protein stability (Kilic et al., 2015a). Functional studies of the *PPARG* rs1801282 polymorphism showed that the presence of risk allele is associated with decreased binding affinity to promoter element, which impacts on ability to transactivate its responsive promoters. This molecular impact could influence adipogenesis and glucose/lipid metabolism, contributing to lower BMI and insulin sensitivity among individuals carrying *PPARG* rs1801282 risk allele (Deeb et al., 1998; Rocha et al., 2015). *UCP2* rs660339 variant influences the reduction of 24-hour-resting energy expenditure and also decreases the fat oxidation rate that could contribute to body weight regulation (Astrup et al., 1999). *ADRB2* rs1042713 polymorphism was shown to reduce thermogenic response to its receptor stimulation, while *ADRB2* rs1042714 did not exhibit the same effect (Oomen et al., 2005). Recently, a functional *in vitro* study of ADRB2 polymorphisms (rs1042713 and rs1042714) showed that mRNA expression was significantly decreased in mutated cells when compared to wild-type (Xie et al., 2019). Our study also chose *DBC1* (rs17060940) and *SIRT1* (rs1467568) polymorphisms. Despite lacking functional studies, these variants or genes have been already investigated or linked to obesity (or related traits) (Garaulet et al., 2012; Jurgens et al., 2022). Therefore, the present study aimed to investigate whether common variants in *DBC1*, *SIRT1*, *PPARG*, *UCP2,* and *ADRB2* genes are potential biomarkers for severe obesity susceptibility and/or metabolic complications in a Brazilian population. These results could provide useful knowledge for clinical management and risk assessment of obesity and/or metabolic dysfunctions.

## Materials and methods

### Study subjects

A case-control cross-sectional study was performed with 501 adult individuals, aged 18—70 years, from Rio de Janeiro, Brazil. The inclusion criteria were participants with normal weight (controls, 18.5≤ BMI ≤24.9 kg/m^2^) and severe obesity (cases, BMI ≥35 kg/m^2^). The exclusion criteria included (1) pregnancy, (2) lactation, and (3) the use of medication to lose or gain weight directly. The case group was recruited from a non-governmental organization, called Rescue Group to Self-Esteem and Citizenship of the Obese (in Portuguese, “Grupo de Resgate à Autoestima e Cidadania do Obeso”). All case participants are candidates for bariatric surgery. The control group consisted of volunteers from public hospitals in the same city. Both cases and controls share a common geographic origin in Southeastern Brazil. All subjects have signed an informed consent form before the enrollment of the study. The protocol of the study was approved by the ethics committee of the Oswaldo Cruz Foundation (CAAE: 09225113.0.0000/Protocol No: 346.634).

### Demographic and lifestyle factors

At the time of recruitment, a standardized questionnaire was applied to each participant by trained interviewers. Race/skin color was self-reported and divided according to the criteria of the Demographic Census conducted through the Brazilian Institute of Geography and Statistics. Marital status was categorized as single, married/cohabiting, separated/divorced, and widower. Smoking habits were assessed as smoking status, divided into “never smoked” or “already smoked” (current smokers and ex-smokers). Physical activity information was classified according to the activities performed in the last month (“yes” or “no”).

### Anthropometric parameters, blood biochemistry, and hormone analysis

All participants were examined after an overnight fast. Body weight, waist, and hip circumference were measured as described earlier (da Fonseca et al., 2019). BMI was calculated as weight (kilograms) divided by the square of height (meters). Body adiposity index (BAI) was calculated by the formula: hip circumference/(height^1.5^)-18. It is used to estimate the pattern of body fat distribution (Bergman et al., 2011). Systolic and diastolic blood pressure was measured in the sitting position using a wrist blood pressure monitor.

Fasting plasma glucose, total cholesterol (TC), high-density lipoprotein cholesterol (HDL-c), and triglyceride (TG) were measured using the oxidase-peroxidase method (BioSystems). Low-density lipoprotein cholesterol (LDL-c) levels were calculated by the Friedewald formula (LDL-c = TC—HDLc—TG/5). C-reactive protein (CRP) and glycated hemoglobin levels were measured by the latex agglutination method and turbidimetric inhibition immunoassay (TINIA), respectively.

Leptin, resistin monocyte chemoattractant protein-1/CCL2 (MCP1), and plasminogen activator inhibitor-1 (PAI-1) were measured using Human Adipocyte Magnetic Bead (Millipore-Merck [cat# HADCYMAG-61k]) on Bio-Plex 200 Multiplexing Analyzer System, according to the manufacturer’s protocol.

### Metabolic health status in obesity

Participants with obesity were classified as balanced metabolic obesity (BMO) and unbalanced metabolic obesity (UMO) according to the presence/absence of metabolic abnormalities. Metabolic status was obtained based on components of metabolic abnormalities: fasting glucose level (≥100 mg/dL or drug treatment for elevated glucose), triglycerides (≥150 mg/dL or drug treatment for elevated triglycerides), HDL-cholesterol (<40 mg/dL in men and <50 mg/dL in women or drug treatment for reduced HDL-cholesterol), and blood pressure (≥130/85 mmHg or drug treatment for hypertension). Participants with equal or less than 1 component of metabolic syndrome were classified as BMO, and equal or more than 2 components were defined as UMO (Grundy et al., 2004; Eckel et al., 2010; Blüher, 2020).

### Molecular analyses

Genomic DNA was extracted from peripheral blood cells using QIAamp Blood Kit (Qiagen, Valencia, CA, United States), according to the manufacturer’s protocol. All samples were genotyped for polymorphisms in *DBC1* (rs17060940), *SIRT1* (rs7895833 and rs1467568), *UCP2* (rs660339), *PPARG* (rs1801282) and *ADRB2* (rs1042713 and rs1042714) genes by real-time PCR using TaqMan^®^ assays (ThermoFisher, Foster City, CA, United States) in a StepOne^®^ Plus Real-Time PCR System (Supplemental Table S1). Allelic discrimination plots were analyzed by StepOne software v2.3.

### Statistical analyses

The normality of continuous variables was analyzed by Kolmogorov–Smirnov and Shapiro–Wilk tests. All data were shown as non-normally dispersed. The distribution of continuous and categorical variables in groups was presented as median (interquartile range) and number (percentage), respectively. Comparison of clinical, anthropometric, biochemical, and plasma biomarkers between cases and controls was performed using Mann-Whitney (quantitative variables) and Chi-square tests (qualitative variables). These statistical analyses were performed using SPSS (SPSS, Chicago, IL, United States). Genotype and allele frequencies were calculated by gene counting. The balance between the genotypes was checked by the Hard-Weinberg Equilibrium (HWE) test. A Pairwise linkage disequilibrium analysis was carried out in *ADRB2* and *SIRT1* variants using Haploview v.4.2. The genetic association was analyzed and controlled for age, gender, skin color, physical activity, and marital status using SNPassoc package (http://CRAN.Rproject.org/package=SNPassoc). The results are shown as odds ratio (OR) with 95% confidence interval (CI). An association analysis between genetic variants and obesity susceptibility was performed under five different genetic models (codominant, dominant, recessive, overdominant and log-additive). The best genetic model was determined using Akaike Information Criteria (AIC) (González et al., 2007). Linear regression analyses were performed to test the association between studied variants and the continuous variables (logarithmically transformed before the test). Age, gender, race/skin color, physical activity, and marital status were used as covariables for body weight and BMI. These variables and BMI were used as confounders for all other continuous parameters studied. The level of significance was set at *p-value* < 0.008 (Bonferroni correction for multiple tests).

According to the Quanto power and sample size calculator, the genetic variants analyzed here have at least 80% power to detect effect sizes between 1.35–1.65, based on the minor allele frequency (MAF) of these alterations (https://keck.usc.edu/biostatistics/software/).

## Results

### Characteristics of the study population

A total of 501 individuals were enrolled in this study, stratified into participants with severe obesity (cases, n = 305) and normal weight (controls, n = 196). The background characteristics of the study subjects are shown in Table 2. As has been known and reported, individuals with severe obesity presented higher anthropometric, blood pressure, and biochemical levels when compared to control participants. In our study, the individuals with normal weight had higher values for height and HDL cholesterol. As expected, individuals with severe obesity had higher levels of leptin, PAI-1, and resistin when compared to controls.

**TABLE 2:** Basic clinical characteristics of the studied population.

Parameters	n	All	n	Control	n	Case	p*
Age (years)	501	36.0 (27.0; 45.0)	196	29.0 (24.0; 37.0)	305	40.0 (31.0; 48.0)	**<0.001**
Gender (female/male)							
Female	501	368 (73.4)	196	121 (61.7)	305	247 (80.9)	**<0.001**
Male		133 (26.6)		75 (38.3)		58 (19.1)	
Race/Skin color							
White	500	236 (47.2)	196	133 (67.8)	304	103 (33.9)	**<0.001**
Brown		89 (17.8)		14 (7.2)		75 (24.7)	
Black		169 (33.8)		49 (25.0)		120 (39.5)	
Others		6 (1.2)		0 (0.0)		6 (1.9)	
Marital status							
Single	499	226 (45.3)	196	122 (62.3)	303	104 (34.3)	**<0.001**
Married/cohabiting		218 (43.7)		63 (32.1)		155 (51.2)	
Separated/divorced		34 (6.8)		4 (2.0)		30 (9.9)	
Widower		21 (4.2)		7 (3.6)		14 (4.6)	
Smoking status							
Already smoked	493	56 (11.3)	195	18 (9.2)	298	38 (12.8)	0.228
Never smoked		437 (88.7)		177 (90.8)		260 (87.2)	
Physical activity practice							
Yes	497	160 (32.2)	196	94 (48.0)	301	243 (80.7)	**<0.001**
No		337 (67.8)		102 (52.0)		58 (19.3)	
Weight (kg)	501	105.0 (67.0; 132.4)	196	62.9 (57.0; 70.0)	305	126.3 (109.2; 144.4)	**<0.001**
Height (m)	501	1.65 (1.59; 1.71)	196		305	1.63 (1.58; 1.69)	**<0.001**
BMI (kg/m^2^)	501	40.1 (23.3; 48.8)	196	22.8 (21.1; 23.9)	305	46.7 (41.8; 52.6)	**<0.001**
Waist circunference (cm)	498	120.0 (84.0; 140.0)	196	81.0 (75.0; 85.5)	302	136.0 (125.0; 147.0)	**<0.001**
Hip circunference (cm)	498	128.0 (99.5; 146.0)	196	98.0 (94.0; 101.5)	302	141.7 (131.5; 153.0)	**<0.001**
WHR	498	0.91 (0.83; 0.98)	196	0.83 (0.78; 0.88)	302	0.96 (0.90; 1.00)	**<0.001**
BAI	493	42.1 (28.1; 51.6)	191	27.0 (24.1; 29.6)	302	49.6 (44.3; 56.1)	**<0.001**
Glucose (mg/dL)	388	93.0 (87.0; 103.0)	175	89.0 (84.0; 95.0)	213	98.0 (90.0; 110.0)	**<0.001**
Total cholesterol (mg/dL)	427	187.0 (161.0; 216.0)	183	179.0 (156.0; 200.0)	244	193.0 (166.2; 224.0)	**<0.001**
HDL cholesterol (mg/dL)	427	51.0 (43.0; 61.0)	183	59.0 (48.0; 69.0)	244	47.0 (41.0; 53.0)	**<0.001**
LDL cholesterol (mg/dL)	420	110.0 (92.0; 135.0)	182	102.5 (86.7; 123.0)	238	118.0 (95.7; 168.0)	**<0.001**
Triglycerides (mg/dL)	427	103.0 (74.0; 139.0)	183	77.0 (61.0; 101.0)	244	125.5 (96.0; 168.0)	**<0.001**
Hemoglobin glycated (%)	342	5.30 (4.30; 5.90)	164	5.10 (4.82; 5.40)	178	5.80 (5.10; 6.32)	**<0.001**
CRP (mg/dL)	338	0.44 (0.12; 1.11)	163	0.14 (0.08; 0.28)	175	1.00 (0.55; 1.55)	**<0.001**
SBP (mm Hg)	355	121.0 (111.0; 134.0)	182	118.0 (110.0; 126.0)	173	130.0 (117.0; 147.5)	**<0.001**
DBP (mm Hg)	355	80.0 (71.0; 90.0)	182	76.0 (68.0; 84.0)	173	85.0 (76.0; 92.0)	**<0.001**
Leptin (pg/mL)	120	2,323.0 (1,141.5; 3,057.7)	35	565.0 (210.7; 887.2)	85	2,656.2 (2,210.6; 3,432.1)	**<0.001**
MCP1 (pg/mL)	120	256.7 (165.5; 363.4)	35	246.6 (140.7; 313.6)	85	258.0 (184.1; 390.2)	0.124
PAI1 (pg/mL)	120	26,233.2 (19,531.7; 31,683.1)	35	22,406.1 (16,573.4; 29,605.9)	85	27,461.2 (20,946.9; 33,222.9)	**0.030**
Resistin (pg/mL)	120	8,370.4 (6,212.7; 10,663.9)	35	7,420.8 (4,673.5; 9,373.2)	85	8,618.1 (6,296.7; 11,190.0)	**0.021**

The bold signal means a statistically significant.

Furthermore, our data showed significant differences in age, gender, color skin/ethnicity, physical activity practice, and marital status between case and control groups. Thus, these five variables were further included as covariates in the logistic and the linear regression model to remove bias from confounding factors (model 2).

### Association analyses of polymorphisms studied and severe obesity risk

The sample details about genotype and allele frequencies are presented in Table 3. *PPARG* rs1801282 polymorphism showed a low frequency in our cohort since the homozygous GG genotype was found only in one control and two case subjects. Additionally, *DBC1* (rs17060940) TT genotype was only observed in the case group (16 individuals). Thus, genetic analyses of *PPARG* rs1801282 and *DBC1* rs17060940 were performed in the dominant model and log-additive. Regarding HWE analyses, only *ADRB2* rs1042713 polymorphism was deviated for controls and was excluded in the following analyses (*p* < 0.05) (Supplemental Table S2). Furthermore, our analysis demonstrated that *SIRT1* (rs7895833 and rs1467568; D’ = 0.973; *r* ^2^ = 0.244; LOD = 37.91) and *ADRB2* (rs1042713 and rs1042714; D’ = 0.829; *r* ^2^ = 0.223; LOD = 31.86) are in linkage disequilibrium.

**TABLE 3:** Association of common variants and severe obesity susceptibility.

Gene (polymorphism)	Control	Case^a^	OR (95% CI)	*p*
	n = 196 (%)	n = 299 (%)		
*DBC1* (rs17060940)				
*Dominant Model*				
CC	153 (78.1)	218 (72.9)	1.00 (Ref.)	-
CT + TT	43 (21.9)	81 (27.1)	1.02 (0.61–1.69)	0.949
Log *additive (0,1,2)*			1.27 (0.81–1.97)	0.292
*SIRT* (rs7895833)				
*Codominant model*				
AA	126 (64.3)	152 (50.8)	1.00 (Ref.)	0.026
AG	62 (31.6)	133 (44.5)	1.84 (1.16–2.90)	
GG	8 (4.1)	14 (4.7)	0.91 (0.30–2.78)	
*Dominant Model*				
AA	126 (64.3)	152 (50.8)	1.00 (Ref.)	-
AG + GG	70 (35.7)	147 (49.2)	1.72 (1.10–2.67)	0.015
*Recessive Model*				
AA + AG	188 (95.9)	285 (95.3)	1.00 (Ref.)	-
GG	8 (4.1)	14 (4.7)	0.72 (0.24–2.17)	0.562
*Overdominant Model*				
AA + GG	134 (68.4)	166 (55.5)	1.00 (Ref.)	-
AG	62 (31.6)	133 (44.5)	1.85 (1.18–2.90)	**0.007**
Log *additive (0,1,2)*			1.45 (0.99–2.13)	0.056
*SIRT* (rs1467568)				
*Codominant model*				
AA	50 (25.5)	108 (36.1)	1.00 (Ref.)	0.159
AG	90 (45.9)	143 (47.8)	0.88 (0.53–1.45)	
GG	56 (28.6)	48 (16.1)	0.56 (0.30–1.04)	
*Dominant Model*				
AA	50 (25.5)	108 (36.1)	1.00 (Ref.)	-
AG + GG	146 (74.5)	191 (63.9)	0.77 (0.48–1.24)	0.280
*Recessive Model*				
AA + AG	140 (71.4)	251 (83.9)	1.00 (Ref.)	-
GG	56 (28.6)	48 (16.1)	0.60 (0.35–1.03)	0.064
*Overdominant Model*				
AA + GG	106 (54.1)	156 (52.2)	1.00 (Ref.)	-
AG	90 (45.9)	143 (47.8)	1.12 (0.72–1.72)	0.620
Log *additive (0,1,2)*			0.76 (0.55–1.03)	0.076
*UCP2 (rs660339)*				
*Codominant model*				
GG	62 (31.6)	80 (26.8)	1.00 (Ref.)	0.171
GA	101 (51.5)	139 (46.5)	1.25 (0.76–2.07)	
AA	33 (16.8)	80 (26.8)	1.81 (0.97–3.39)	
*Dominant Model*				
GG	62 (31.6)	80 (26.8)	1.00 (Ref.)	-
GA + AA	134 (68.4)	219 (73.2)	1.40 (0.87–2.25)	0.169
*Recessive Model*				
GG + GA	163 (83.2)	219 (73.2)	1.00 (Ref.)	-
AA	33 (16.8)	80 (26.8)	1.57 (0.92–2.69)	0.097
*Overdominant Model*				
GG + AA	95 (48.5)	160 (53.5)	1.00 (Ref.)	-
GA	101 (51.5)	139 (46.5)	0.98 (0.63–1.50)	0.911
Log *additive (0,1,2)*			1.34 (0.98–1.82)	0.064
*PPARG (rs1801282)*				
*Dominant Model*				
CC	178 (90.8)	250 (83.6)	1.00 (Ref.)	-
CG + GG	18 (9.2)	49 (16.4)	2.29 (1.18–4.45)	0.012
Log *additive (0,1,2)*			2.06 (1.08–3.91)	0.023
*ADRB2 (rs1042714)*				
*Codominant model*				
CC	98 (50.0)	138 (46.2)	1.00 (Ref.)	0.216
CG	75 (38.3)	126 (42.1)	1.49 (0.94–2.37)	
GG	23 (11.7)	35 (11.7)	1.39 (0.67–2.88)	
*Dominant Model*				
CC	98 (50.0)	138 (46.2)	1.00 (Ref.)	-
CG + GG	98 (50.0)	161 (53.8)	1.47 (0.95–2.28	0.082
*Recessive Model*				
CC + CG	173 (88.3)	264 (88.3)	1.00 (Ref.)	-
GG	23 (11.7)	35 (11.7)	1.15 (0.58–2.30)	0.687
*Overdominant Model*				
CC + GG	121 (61.7)	173 (57.9)	1.00 (Ref.)	-
CG	75 (38.3)	126 (42.1)	1.40 (0.92–1.77)	0.133
Log *additive (0,1,2)*			1.28 (0.92–1.77)	0.138

Note.

^a^ Six participants were not included in the analysis since they did not have covariates information to adjust. The bold signal means a statistically significant.

The results of our case-control association analyses are also shown in Table 3. The genotype frequencies of *SIRT1* rs7895833 differed significantly between case and control groups, indicating that this polymorphism is associated with severe obesity risk. The frequency of *SIRT1* rs7895833 AG genotype was higher in the case group when compared to the control (44.5% vs. 31.6%, respectively). To explore this association, dominant, recessive, overdominant and log-additive models were carried out. *SIRT1* rs7895833 was associated with severe obesity in overdominant model. In this line, overdominant was the lowest AIC score. The *SIRT1* rs7895833 AG was observed as a genetic predisposition factor for severe obesity development, increasing the risk more than 1.8 times [OR = 1.85 (1.18–2.90); *p* = 0.007] when compared to GG + AA genotypes. No association was observed between severe obesity and *DBC1* (rs17060940), *SIRT1* (rs1467568), *PPARG* (rs1801282), *UCP2* (rs660339), and *ADRB2* (rs1042714).

### Comparisons of metabolic variables according to polymorphisms studied

The effect of the studied polymorphisms on metabolic variables was also investigated in our sample (Supplemental Table S3). *DBC1* rs17060940 was associated with glycated hemoglobin, in which the presence of a mutated allele was associated with higher levels. *SIRT1* rs1467568 was associated with BAI and HC, in which the median values decreased according to the number of risk alleles. Thus, carriers of two mutated alleles (AA) had lower median values when compared to none or one. Finally, *UCP2* rs660339 influenced BMI. Interestingly, our results highlighted that the *UCP2* rs660339 AA genotype had an increased BMI median when compared to GG or GA genotypes. None of the other common variants studied was seen as related to the metabolic variables. We also did not observe an association between the polymorphisms studied and the levels of adipokines and inflammatory markers.

### Metabolic health status and association analyses of polymorphisms and hormones studied

Metabolic health status was obtained in 256 participants with obesity, in which 27.3% were BMO and 72.7% were UMO. Interestingly, our data indicated that individuals with UMO have increased levels of leptin when compared to BMO (2,830.5 [2,238.5; 3,812.1] vs. 2,350.6 [1,745.5; 2,656.2], respectively) (*p* = 0.017). However, this result did not remain after correcting for multiple tests. No significant results were observed with MCP1, PAI1, and resistin (data not shown). In addition, our results indicated that *SIRT1* rs1467568 was associated with metabolic health status in our cohort (Table 4). The frequency of the *SIRT1* rs1467568 AG genotype was higher in the UMO group when compared to BMO (53.8% vs. 32.9%, respectively). To deeply investigate this association, different genetic models were performed. Our results indicated that *SIRT1* rs1467568 was associated with metabolic health status in overdominant models. Individuals carrying the heterozygous genotype (AG) were 2.5 times more likely to develop UMO when compared to GG + AA [OR = 2.49 (1.36—4.56); *p* = 0.003]. No association was observed between metabolic healthy status and *DBC1* (rs17060940), *SIRT* (rs7895833), *UCP2* (rs660339), *PPARG* (rs1801282), and *ADRB2* (rs1042714) (data not shown).

**TABLE 4:** Association of *SIRT* rs1467568 polymorphism and metabolic status.

Gene (polymorphism)	BMO	UMO		
	n = 70	n = 186	OR (95% CI)	*p*
*SIRT* (rs1467568)				
*Genotype*				
AA	32 (45.7)	61 (32.8)	1.00 (Ref.)	-
AG	23 (32.9)	100 (53.8)	2.21 (1.16–4.25)	0.017
GG	15 (21.4)	25 (13.4)	0.66 (0.29–1.15)	0.324
*Dominant Model*				
AA	32 (45.7)	61 (32.8)	1.00 (Ref.)	-
AG + GG	38 (54.3)	125 (67.2)	1.59 (0.88–2.85)	0.123
*Recessive Model*				
AA + AG	55 (78.6)	161 (86.6)	1.00 (Ref.)	-
GG	15 (21.4)	25 (13.4)	0.44 (0.20–0.94)	0.035
*Overdominant Model*				
AA + GG	47 (67.1)	86 (46.2)	1.00 (Ref.)	-
AG	23 (32.9)	100 (53.8)	2.49 (1.36–4.56)	**0.003**
*Allele*				
A	87 (62.0)	222 (60.0)	1.00 (Ref.)	-
G	53 (38.0)	150 (40.0)	0.95 (0.61–1.46)	0.820

The bold signal means a statistically significant.

## Discussion

Obesity is a serious public health issue, and understanding its genetic background has gained importance in the world. Several studies have led to the identification of genes that contribute to obesity; however, only a small fraction of its genetic heritability has been elucidated. Additionally, obesity is considered a risk factor for metabolic disorders, although there is a large variation in the individual risk of developing these alterations. Our study focused on the molecular pathway involved in adipose tissue fat capacity and inflammation to investigate potential genetic biomarkers for severe obesity and metabolic health status in obesity. To this end, we performed a Brazilian case-control study and genotyped subjects with severe obesity (n = 305) and normal-weight (n = 196) for common variants in *DBC1*, *SIRT1*, *UCP2*, *PPARG* and *ADRB2* genes. We also measured important adipokines and hormones classically linked to obesity, such as leptin, MCP1, PAI-1, and resistin, in order to characterize our sample as well as to evidence possible associations between the variants and the secretion of these mediators.

Despite this important role in adipogenesis and body metabolic function, information about the association between *DBC1* polymorphisms and obesity is lacking in the literature. Our study investigated a splice polypyrimidine tract variant in *DBC1* gene (rs17060940), which may disrupt pre-mRNA splicing. This disruption may lead to exon skipping or partial/full intron retention in the mature mRNA, altering the protein-coding sequence (Dufner-Almeida et al., 2019). Our results showed that the presence of the mutant (T) allele does not *per se* confer a risk for severe obesity in our population. However, the mutant genotype TT was found only in our case group (n = 16) with a frequency of 5.1%. According to the 1000 Genomes Project (Phase 3), the frequency of genotype TT varies with the population. The highest frequency is found in Africa (15.3%), followed by South Asia (0.4%) and America (0.3%). This genotype was not observed in Europe and East Asia. In the Online Archive of Brazilian Mutations (ABraOM) database, the frequency of genotype TT was 10.8% (available at http://abraom.ib.usp.br/). Unfortunately, no clinical data is available in this database. Thus, we suggest that this variant should be investigated in other studies using cohorts from different populations to examine the possible influence on obesity susceptibility.

In our analysis, the presence of mutant allele (T) in *DBC1* rs17060940 was significantly associated with higher levels of glycated hemoglobin. Previous studies have suggested that *DBC1* regulates the metabolic gene network and it is downregulated in cells of patients with T2DM (Sathishkumar et al., 2016; Basu et al., 2020). Briefly, DBC1 acts as a negative regulator of histone deacetylase 3 (HDAC3), which is responsible for controlling inflammation and the metabolism of glucose and insulin. It was also reported that the HDAC3 activity/HDAC3 mRNA levels are positively linked to inflammation, poor glycemic control (higher levels of fasting plasma glucose and glycated hemoglobin), and insulin resistance (Sathishkumar et al., 2016). Based on the literature, we suggest that *DBC1* rs17060940 polymorphism may impact the structure of DBC1 pre-mRNA, decreasing the levels of the transcript. This reduction may affect the negative regulation of HDAC3 that impacts glycemic control, consequently, it increases the levels of glycated hemoglobin. This hypothesis is in line with previous studies, which reported that variants in *DBC1* were associated with diabetes risk in the United Kingdom Biobank. Furthermore, polymorphisms near this gene were also linked to diabetes susceptibility (Jurgens et al., 2022).

SIRT1 is involved in biological processes of inflammation as well as glucose and fat metabolism by regulating the transcriptional activity of downstream genes (Picard et al., 2004; Bordone et al., 2006; Chaudhary and Pfluger, 2009; Kotas et al., 2013; Moreno-Navarrete et al., 2015). Our study has investigated two variants in *SIRT1*, which were associated with obesity susceptibility and/or obesity traits (Zillikens et al., 2009; Shimoyama et al., 2011; Kilic et al., 2015b; Higashibata et al., 2016; Casarotto et al., 2019). *SIRT1* rs7895833 (A>G) is located in the promoter region, specifically, it lies in a W-box-like element (sequence TTGACT) (Hwang et al., 2008; Tao et al., 2022). In our research, the *SIRT1* rs7895833 AG heterozygous genotype increased the risk by 1.93 times for severe obesity, when compared to the homozygous forms (AA and GG) [overdominant model].

In a previous study, the *SIRT1* rs7895833 AG genotype carried a high risk of obesity in children from Turkey—as shown in our study. They also suggested that the A allele has a protective effect against obesity (Kilic et al., 2015b). According to a Dutch study, *SIRT1* rs7895833 A allele was associated with higher BMI (Zillikens et al., 2009). In Japanese, the A allele increased the risk for obesity in male individuals (Shimoyama et al., 2011). Further, this allele was also a risk factor for overweight women who did not restrict their calorie intake. However, it was not correlated with BMI in women who controlled their calorie intake or practiced physical exercises (Higashibata et al., 2016). Finally, *SIRT1* rs7895833 polymorphism was not associated with obesity in Brazilian adults (MENEGUETTE et al., 2016). These different findings may be explained by a study design, sample criteria selection, and genetic background.

In the literature, it has been described that the variants in the promoter region are more common than in coding regions of a gene and may affect its expression (Deihimi et al., 2012). The *SIRT1* rs7895833 polymorphism lies on a W-box-like element in the promoter region, which might disturb the gene expression. Based on the literature, SIRT1 negatively controls adipogenesis by repressing genes associated with white adipocyte differentiation and fat accumulation. Moreover, SIRT1 interacts with PPARG to suppress its transcriptional activity, which results in the inhibition of adipogenesis (Picard et al., 2004). In this context, a previous study also observed that lower SIRT1 expression is associated with obesity (Clark et al., 2012). We hypothesized that the *SIRT1* rs7895833 polymorphism may affect the promoter activity, decreasing the expression of the *SIRT1* gene. Consequently, it may impact SIRT1 negative regulation, increasing the risk for obesity. However, functional studies are necessary to confirm this hypothesis.

Concerning the *SIRT1* rs1467568 polymorphism, the presence of a minor allele (G) was associated with lower BAI and HC in our study. Our analyses showed a dose-effect relationship between the polymorphism and these anthropometric traits. This variant located in the intronic region was previously associated with obesity and weight loss resistance in a therapy treatment (Zillikens et al., 2009; Clark et al., 2012; Garaulet et al., 2012). Our findings are in line with previous studies which reported that the *SIRT1* rs1467568 G allele has a protective effect against obesity in a Dutch and French population (Zillikens et al., 2009; Clark et al., 2012). Besides these results, we also observed that the *SIRT1* rs1467568 AG genotype is a risk factor for UMO in individuals with severe obesity. To our knowledge, no study has investigated this association until now. However, it was reported that ∼10–30% of subjects with obesity have a lower risk for cardiometabolic abnormalities (Blüher, 2020)—similar to our findings. Phenome-wide association study (PheWAS) was used to deeply investigate the effect of *SIRT1* rs1467568 on anthropometric, biochemical, and blood pressure in the literature. This variant was associated with BMI, weight, glucose, glycated hemoglobin, HOMA-IR as well as systolic and diastolic blood pressure (https://gwas.mrcieu.ac.uk/phewas/). Thus, we suggest that more studies should investigate this association to understand the genetic background of cardiometabolic abnormalities.

The *PPARG* plays an important role in adipogenesis, and glucose and lipid metabolism (Spiegelman, 1998; Auwerx, 1999). The *PPARG* rs1801282 is one of the most studied variants in this gene, which leads to the replacement of alanine with proline at codon 12 [p. (Pro12Ala)]. Previous studies reported an association of this variant with obesity risk and measures of this disease; however, others have reported contradictory results (Masud et al., 2003; Ali et al., 2009; Carlos et al., 2013; Hsiao and Lin, 2015; Hasan et al., 2017; Bakhashab et al., 2020; Dmitrenko et al., 2022). Our study showed that *PPARG* rs1801282 was not associated with severe obesity risk nor influenced obesity traits variables. Our findings are in line with Bakhashab and coworkers (2020) who reported no association between *PPARG* rs1801282 and anthropometric/biochemical parameters (BMI, HbA1c, FBS, triglyceride and cholesterol) in individuals from the Saudi population (Bakhashab et al., 2020).

Different findings were observed by Prakash and coworkers (2012), which reported that rs1801282 was associated with obesity risk (dominant model) in the North Indian population (Prakash et al., 2012). Similar results were also observed in two Caucasian populations (Beamer et al., 1998). A previous meta-analysis study observed that the mutant allele (G) was associated with higher BMI in individuals with overweight/obesity. They also found that BMI was 0.07 units higher in individuals carrying this allele (Masud et al., 2003). Ali and colleagues (2009) observed a gender-specific effect of this polymorphism with on obesity risk. They reported that the presence of mutant allele (G) was a risk factor for obesity in a male Tunisian population (Ali et al., 2009). These results are in agreement with Carlos and coworkers (2013), which did not observe an association between *PPARG* rs1801282 and obesity in Portuguese women (Carlos et al., 2013). However, Hsiao and colleagues (2015) reported that *PPARG* rs1801282 predicts overweight and higher BMI only in females from the Taiwan population (Hsiao and Lin, 2015). In a Brazilian cohort, it was also found that this polymorphism was associated with overweight and obesity only in female individuals. Despite our different results, they also observed a low frequency of *PPARG* rs1801282 GG genotype in the case (1.0%) and control group (0.80%) (Castro et al., 2021).

*UCP2* has a crucial role in the energy metabolism of cells. *UCP2* is a candidate gene for obesity and some common variants have been investigated. One of these variants is the missense polymorphism in exon 4 (rs660339), which changes an alanine to valine in codon 55 [p. (Ala55Val)] (Nicoletti et al., 2017). In a number of genetic studies, the association between *UCP2* rs660339 polymorphism and obesity risk has been investigated. However, the results of these studies have been controversial: while some of them reported an association, others were unable to find any relationship between this variant and obesity susceptibility (Kubota et al., 1998; Xiu et al., 2004; Yu et al., 2005; Saraswati et al., 2011; Qian et al., 2013). Previous studies reported that the *UCP2* rs660339 AA genotype increased the risk for obesity development, and it was associated with higher BMI or abdominal obesity when compared to subjects with GG and GA genotypes from China and North America (Xiu et al., 2004; Yu et al., 2005). These results are similar to our findings, in which AA genotype was associated with higher BMI. However, such results were not found in a cohort of Japanese and Indonesian individuals (Kubota et al., 1998; Saraswati et al., 2011). Similarly, a meta-analysis study also showed that this polymorphism was not associated with BMI in subjects of Asian and European descent (Qian et al., 2013). Thus, all these findings suggest that the effect of *UCP2* rs660339 on obesity susceptibility is dependent on the ethnic group.

*ADRB2* is involved in lipid mobilization, especially in adipocyte cells. The missense variant *ADRB2* rs1042714 is one of the most common polymorphisms in this gene, leading to the alteration of glutamine to glutamic acid at codon 27 [p. (Gln27Glu)]. Due to the modification of the amino acid sequence in the extracellular N-terminus of the protein, it was suggested that this polymorphism may alter its function (Reihsaus et al., 1993; Zhang et al., 2014). In the literature, the results concerning the contribution of *ADRB2* rs1042714 polymorphism toward obesity development (or obesity-traits) are contradictory. Two previous meta-analysis studies involving this polymorphism and obesity highlighted that these different results may be explained due to the divergence in the genetic background. First, Jalba and coworkers (2008) reported that *ADRB2* rs1042714 is a risk factor for obesity in Asians, Pacific Islanders, and American Indians, but not in Europeans (Jalba et al., 2008). Later, Zhang and colleagues (2014) conducted a meta-analysis that involved 17 studies (n = 9,995 subjects), in which *ADRB2* rs1042714 was associated with increased obesity susceptibility in the heterozygote and dominant model (Zhang et al., 2014). The results of our study showed no significant association between *ADRB2* rs1042714 and obesity, which was similar to those observed in Europeans (Jalba et al., 2008). Our results supported previous results of Kortner (Kortner et al., 1999), Galletti (Galletti et al., 2004) and Oberkofler (Oberkofler et al., 2000), which reported no association in a German, Italian and Australian populations—respectively.

Regarding the adipokines and/or inflammatory markers, our results showed that individuals with obesity have higher levels of leptin, PAI-1, and resistin. These results are in accordance with previous reports in the literature. It is well-established obesity alters secretory adipose tissue function in rodents and humans, especially adipokines and pro-inflammatory mediator secretions (Codoñer-Franch and Alonso-Iglesias, 2015; Barnard et al., 2016; Obradovic et al., 2021).

PAI-1 is produced by adipose tissue and is found to increase in individuals with obesity, being suggested as a biochemical marker of this disease. Additionally, PAI-1 is a component of metabolic syndrome and it is the main inhibitor of fibrinolysis, reenforcing to be a risk factor for cardiovascular disease in individuals with obesity (Mertens et al., 2006; Barnard et al., 2016). Resistin is also an important factor in obesity-mediated inflammation and plays a key role as a trigger of metabolic complications and insulin resistance (Benomar and Taouis, 2019). Previous studies showed that resistin is a key hormone linking hypertension and insulin resistance to obesity through activating the Toll-Like Receptor (TLR4) pathway, which can promote the progressive onset of type 2 diabetes (Benomar et al., 2016; Jiang et al., 2016; Benomar and Taouis, 2019). Finally, leptin is a hormone that regulates food intake, total body fat, and proinflammatory immune responses. It is produced and secreted by white adipose tissue, and smaller quantities are detected in brown adipose tissue (MacDougald et al., 1995). Regarding the molecular effect of leptin, this adipokine promotes the expression of several inflammatory cytokines. In a feedback loop, these inflammatory makers increase leptin expression, promoting a low-grade and chronic inflammation (Dubern and Clement, 2012; Obradovic et al., 2021).

Our study has limitations that have to be considered. (1) It was a cross-sectional study: we were not able to eliminate fluctuations in clinical, anthropometric and biochemical parameters. (2) different variables were included as confounding factors; however, we cannot exclude the complete role of environmental variables, which could influence the severe obesity risk. (3) It was not possible to confirm that the studied polymorphisms affect gene expression since we do not have data on mRNA or protein expression.

In conclusion, our data present putative genetic biomarkers for severe obesity and metabolic complications susceptibility. Our findings showed that *SIRT1* rs7895833 (AG) increased the risk for severe obesity, and *SIRT1* rs1467568 (AG) was associated with UMO development. We also observed that *SIRT1* rs1467568, *DBC1* rs17060940, and *UCP2* rs660339 influenced obesity-related traits in the studied Brazilian population. Therefore, our results indicated that *SIRT1* rs7895833 and *SIRT1* rs1467568 may be genetic biomarkers for severe obesity and/or unbalanced metabolic status.

## Data Availability

The original contributions presented in the study are included in the article/Supplementary Material, further inquiries can be directed to the corresponding author.

## References

[B1] Abarca-Gómez L. Abdeen Z. A. Hamid Z. A. Abu-Rmeileh N. M. Acosta-Cazares B. Acuin C. (2017). Worldwide trends in body-mass index, underweight, overweight, and obesity from 1975 to 2016: a pooled analysis of 2416 population-based measurement studies in 128·9 million children, adolescents, and adults. Lancet 390 (10113), 2627–2642. 10.1016/S0140-6736(17)32129-3 29029897 PMC5735219

[B2] Affourtit C. Brand M. D. (2008). On the role of uncoupling protein-2 in pancreatic beta cells. Biochim. Biophys. Acta - Bioenerg. 1777 (7), 973–979. 10.1016/j.bbabio.2008.03.022 18433713

[B3] Ali S. B. Yahia F. B. Sediri Y. Kallel A. Ftouhi B. Feki M. (2009). Gender-specific effect of Pro12Ala polymorphism in peroxisome proliferator-activated receptor γ-2 gene on obesity risk and leptin levels in a Tunisian population. Clin. Biochem. 42 (16–17), 1642–1647. 10.1016/j.clinbiochem.2009.08.019 19733160

[B4] Almandoz J. P. Singh E. Howell L. A. Grothe K. Vlazny D. T. Smailovic A. (2013). Spillover of fatty acids during dietary fat storage in type 2 diabetes: relationship to body fat depots and effects of weight loss. Diabetes 62 (6), 1897–1903. 10.2337/db12-1407 23349503 PMC3661646

[B5] Astrup A. Toubro S. Dalgaard L. T. Urhammer S. A. Sørensen T. I. A. Pedersen O. (1999). Impact of the v/v 55 polymorphism of the uncoupling protein 2 gene on 24‐h energy expenditure and substrate oxidation. Int. J. Obes. 23 (10), 1030–1034. 10.1038/sj.ijo.0801040 10557023

[B6] Auwerx J. (1999). PPARgamma, the ultimate thrifty gene. Diabetologia 42 (9), 1033–1049. 10.1007/s001250051268 10447513

[B7] Azzu V. Brand M. D. (2010). The on-off switches of the mitochondrial uncoupling proteins. Trends Biochem. Sci. 35 (5), 298–307. 10.1016/j.tibs.2009.11.001 20006514 PMC3640847

[B8] Bakhashab S. Filimban N. Altall R. M. Nassir R. Qusti S. Y. Alqahtani M. H. (2020). The effect sizes of PPARγ rs1801282, FTO rs9939609, and MC4R rs2229616 variants on type 2 diabetes mellitus risk among the western Saudi population: a cross-sectional prospective study. Genes (Basel) 11 (1), 98. 10.3390/genes11010098 31947684 PMC7017045

[B9] Barnard S. A. Pieters M. De Lange Z. (2016). The contribution of different adipose tissue depots to plasma plasminogen activator inhibitor-1 (PAI-1) levels. Blood Rev. 30 (6), 421–429. 10.1016/j.blre.2016.05.002 27233154

[B10] Basu S. Barad M. Yadav D. Nandy A. Mukherjee B. Sarkar J. (2020). DBC1, p300, HDAC3, and Siah1 coordinately regulate ELL stability and function for expression of its target genes. Proc. Natl. Acad. Sci. 117 (12), 6509–6520. 10.1073/pnas.1912375117 32152128 PMC7104407

[B11] Beamer B. A. Yen C.-J. Andersen R. E. Muller D. Elahi D. Cheskin L. J. (1998). Association of the Pro12Ala variant in the peroxisome proliferator-activated receptor-gamma2 gene with obesity in two Caucasian populations. Diabetes 47 (11), 1806–1808. 10.2337/diabetes.47.11.1806 9792554

[B12] Benomar Y. Amine H. Crépin D. Al Rifai S. Riffault L. Gertler A. (2016). Central resistin/TLR4 impairs adiponectin signaling, contributing to insulin and FGF21 resistance. Diabetes 65 (4), 913–926. 10.2337/db15-1029 26740596

[B13] Benomar Y. Taouis M. (2019). Molecular mechanisms underlying obesity-induced hypothalamic inflammation and insulin resistance: pivotal role of resistin/TLR4 pathways. Front. Endocrinol. (Lausanne) 10, 140. 10.3389/fendo.2019.00140 30906281 PMC6418006

[B14] Bergman R. N. Stefanovski D. Buchanan T. A. Sumner A. E. Reynolds J. C. Sebring N. G. (2011). A better index of body adiposity. Obesity 19 (5), 1083–1089. 10.1038/oby.2011.38 21372804 PMC3275633

[B15] Blüher M. (2020). Metabolically healthy obesity. Endocr. Rev. 41 (3), bnaa004. 10.1210/endrev/bnaa004 32128581 PMC7098708

[B16] Bordone L. Motta M. C. Picard F. Robinson A. Jhala U. S. Apfeld J. (2006). Sirt1 regulates insulin secretion by repressing UCP2 in pancreatic beta cells. PLoS Biol. 4 (2), e31. 10.1371/journal.pbio.0040031 16366736 PMC1318478

[B17] Carlos F. F. Silva-Nunes J. Flores O. Brito M. Doria G. Veiga L. (2013). Association of FTO and PPARG polymorphisms with obesity in Portuguese women. Diabetes, Metab. Syndr. Obes. targets Ther. 6, 241–245. 10.2147/DMSO.S45779 PMC3712741 23874114

[B18] Casarotto A. A. F. Galera B. B. Sumiyoshi L. M. Floôr T. M. (2019). Polymorphism rs7895833 in the SIRT1 gene and its association with dyslipidaemia in the elderly. Rev. Esp. Geriatr. Gerontol. 54 (4), 214–219. 10.1016/j.regg.2019.01.008 31040057

[B19] Castro G. V. Latorre A. F. S. Korndorfer F. P. de Carlos Back L. K. Lofgren S. E. (2021). The impact of variants in four genes: MC4R, FTO, PPARG and PPARGC1A in overweight and obesity in a large sample of the Brazilian population. Biochem. Genet. 59 (6), 1666–1679. 10.1007/s10528-021-10079-2 34057646

[B20] Chaudhary N. Pfluger P. T. (2009). Metabolic benefits from Sirt1 and Sirt1 activators. Curr. Opin. Clin. Nutr. Metab. Care 12 (4), 431–437. 10.1097/MCO.0b013e32832cdaae 19474719

[B21] Clark S. J. Falchi M. Olsson B. Jacobson P. Cauchi S. Balkau B. (2012). Association of sirtuin 1 (SIRT1) gene SNPs and transcript expression levels with severe obesity. Obesity 20 (1), 178–185. 10.1038/oby.2011.200 21760635 PMC3760128

[B22] Codoñer-Franch P. Alonso-Iglesias E. (2015). Resistin: insulin resistance to malignancy. Clin. Chim. acta. 438, 46–54. 10.1016/j.cca.2014.07.043 25128719

[B23] da Fonseca A. C. P. Abreu G. M. Zembrzuski V. M. Campos Junior M. Carneiro J. R. I. Neto J. F. N. (2019). The association of the fat mass and obesity-associated gene (FTO) rs9939609 polymorphism and the severe obesity in a Brazilian population. Diabetes, Metab. Syndr. Obes. Targets Ther. 12, 667–684. 10.2147/DMSO.S199542 PMC6537458 31213864

[B24] da Fonseca A. C. P. Mastronardi C. Johar A. Arcos-Burgos M. Paz-Filho G. (2017). Genetics of non-syndromic childhood obesity and the use of high-throughput DNA sequencing technologies. J. Diabetes Complicat. 31 (10), 1549–1561. 10.1016/j.jdiacomp.2017.04.026 28735903

[B25] Deeb S. S. Fajas L. Nemoto M. Pihlajamäki J. Mykkänen L. Kuusisto J. (1998). A Pro12Ala substitution in PPARgamma2 associated with decreased receptor activity, lower body mass index and improved insulin sensitivity. Nat. Genet. 20 (3), 284–287. 10.1038/3099 9806549

[B26] Deihimi T. Niazi A. Ebrahimi M. Kajbaf K. Fanaee S. Bakhtiarizadeh M. R. (2012). Finding the undiscovered roles of genes: an approach using mutual ranking of coexpressed genes and promoter architecture-case study: dual roles of thaumatin like proteins in biotic and abiotic stresses. Springerplus 1 (1), 30–10. 10.1186/2193-1801-1-30 23961360 PMC3725900

[B27] Dmitrenko O. P. Karpova N. S. Nurbekov M. K. (2022). ACE, PPARG, SIRT1 gene polymorphisms but not PPARGC1A polymorphism are risk factors for gestational diabetes in the Russian population. J. Genet. Genomic Sci. 7 (035), 2. 10.24966/GGS-2485/100035

[B28] Dubern B. Clement K. (2012). Leptin and leptin receptor-related monogenic obesity. Biochimie 94 (10), 2111–2115. 10.1016/j.biochi.2012.05.010 22627381

[B29] Dufner-Almeida L. G. do Carmo R. T. Masotti C. Haddad L. A. (2019). in Chapter Two - understanding human DNA variants affecting pre-mRNA splicing in the NGS era (United States: Academic Press), 39–90. 10.1016/bs.adgen.2018.09.002 30904096

[B30] Eckel R. H. Alberti KGMM Grundy S. M. Zimmet P. Z. (2010). The metabolic syndrome. Lancet 375 (9710), 181–183. 10.1016/S0140-6736(09)61794-3 20109902

[B31] Escande C. Nin V. Pirtskhalava T. Chini C. C. S. Tchkonia T. Kirkland J. L. (2015). Deleted in breast cancer 1 limits adipose tissue fat accumulation and plays a key role in the development of metabolic syndrome phenotype. Diabetes 64 (1), 12–22. 10.2337/db14-0192 25053585 PMC4274806

[B32] Galletti F. Iacone R. Ragone E. Russo O. Valle E. D. Siani A. (2004). Lack of association between polymorphism in the beta2-adrenergic receptor gene, hypertension, and obesity in the Olivetti heart study. Am. J. Hypertens. 17 (8), 718–720. 10.1016/j.amjhyper.2004.04.012 15323067

[B33] Garaulet M. Esteban Tardido A. Lee Y. C. Smith C. E. Parnell L. D. Ordovas J. M. (2012). SIRT1 and CLOCK 3111T> C combined genotype is associated with evening preference and weight loss resistance in a behavioral therapy treatment for obesity. Int. J. Obes. 36 (11), 1436–1441. 10.1038/ijo.2011.270 PMC4428942 22310473

[B34] González J. R. Armengol L. Solé X. Guinó E. Mercader J. M. Estivill X. (2007). SNPassoc: an R package to perform whole genome association studies. Bioinformatics 23 (5), 644–645. 10.1093/bioinformatics/btm025 17267436

[B35] Grundy S. M. Brewer H. B. Cleeman J. I. Smith S. C. Lenfant C. American Heart Association (2004). Definition of metabolic syndrome: report of the national heart, lung, and blood Institute/American heart association conference on scientific issues related to definition. Circulation 109 (3), 433–438. 10.1161/01.CIR.0000111245.75752.C6 14744958

[B36] Hasan N. S. Kamel S. A. Hamed M. Awadallah E. Rahman A. H. A. Musa N. I. (2017). Peroxisome proliferator-activated receptor-γ polymorphism (rs1801282) is associated with obesity in Egyptian patients with coronary artery disease and type 2 diabetes mellitus. J. Genet. Eng. Biotechnol. 15 (2), 409–414. 10.1016/j.jgeb.2017.08.002 30647679 PMC6296640

[B37] Higashibata T. Wakai K. Naito M. Morita E. Hishida A. Hamajima N. (2016). Effects of self-reported calorie restriction on correlations between SIRT1 polymorphisms and body mass index and long-term weight change. Gene 594 (1), 16–22. 10.1016/j.gene.2016.08.051 27591970

[B38] Hsiao T.-J. Lin E. (2015). The Pro12Ala polymorphism in the peroxisome proliferator-activated receptor gamma (PPARG) gene in relation to obesity and metabolic phenotypes in a Taiwanese population. Endocrine 48 (3), 786–793. 10.1007/s12020-014-0407-7 25182148

[B39] Hwang S.-H. Lee I. A. Yie S. W. Hwang D.-J. (2008). Identification of an OsPR10a promoter region responsive to salicylic acid. Planta 227 (5), 1141–1150. 10.1007/s00425-007-0687-8 18193274 PMC2270913

[B40] Jalba M. S. Rhoads G. G. Demissie K. (2008). Association of codon 16 and codon 27 beta 2-adrenergic receptor gene polymorphisms with obesity: a meta-analysis. Obesity 16 (9), 2096–2106. 10.1038/oby.2008.327 19186333

[B41] Jiang Y. Lu L. Hu Y. Li Q. An C. Yu X. (2016). Resistin induces hypertension and insulin resistance in mice via a TLR4-dependent pathway. Sci. Rep. 6 (1), 22193. 10.1038/srep22193 26917360 PMC4768137

[B42] Jurgens S. J. Choi S. H. Morrill V. N. Chaffin M. Pirruccello J. P. Halford J. L. (2022). Analysis of rare genetic variation underlying cardiometabolic diseases and traits among 200,000 individuals in the UK Biobank. Nat. Genet. 54 (3), 240–250. 10.1038/s41588-021-01011-w 35177841 PMC8930703

[B44] Kilic U. Gok O. Elibol-Can B. Ozgen I. T. Erenberk U. Uysal O. (2015b). SIRT1 gene variants are related to risk of childhood obesity. Eur. J. Pediatr. 174 (4), 473–479. 10.1007/s00431-014-2424-1 25233986

[B45] Kilic U. Gok O. Erenberk U. Dundaroz M. R. Torun E. Kucukardali Y. (2015a). A remarkable age-related increase in SIRT1 protein expression against oxidative stress in elderly: SIRT1 gene variants and longevity in human. PLoS One 10 (3), e0117954. 10.1371/journal.pone.0117954 25785999 PMC4365019

[B46] Kortner B. Wolf A. Wendt D. Beisiegel U. Evans D. (1999). Lack of association between a human beta-2 adrenoceptor gene polymorphism (gln27glu) and morbid obesity. Int. J. Obes. 23 (10), 1099–1100. 10.1038/sj.ijo.0801063 10557032

[B47] Kotas M. E. Gorecki M. C. Gillum M. P. (2013). Sirtuin-1 is a nutrient-dependent modulator of inflammation. Adipocyte 2 (2), 113–118. 10.4161/adip.23437 23805409 PMC3661114

[B48] Kubota T. Mori H. Tamori Y. Okazawa H. Fukuda T. Miki M. (1998). Molecular screening of uncoupling protein 2 gene in patients with noninsulin-dependent diabetes mellitus or obesity. J. Clin. Endocrinol. Metab. 83 (8), 2800–2804. 10.1210/jcem.83.8.4994 9709950

[B49] Kurylowicz A. Jonas M. Lisik W. Jonas M. Wicik Z. A. Wierzbicki Z. (2015). Obesity is associated with a decrease in expression but not with the hypermethylation of thermogenesis-related genes in adipose tissues. J. Transl. Med. 13 (1), 31. 10.1186/s12967-015-0395-2 25622596 PMC4314800

[B50] Lafontan M. Sengenes C. Galitzky J. Berlan M. De Glisezinski I. Crampes F. (2000). Recent developments on lipolysis regulation in humans and discovery of a new lipolytic pathway. Int. J. Obes. 24 (4), S47–S52. 10.1038/sj.ijo.0801505 11126242

[B51] Lê K.-A. Mahurkar S. Alderete T. L. Hasson R. E. Adam T. C. Kim J. S. (2011). Subcutaneous adipose tissue macrophage infiltration is associated with hepatic and visceral fat deposition, hyperinsulinemia, and stimulation of NF-κB stress pathway. Diabetes 60 (11), 2802–2809. 10.2337/db10-1263 22025778 PMC3198061

[bib83] Lee M. Choi S. Lee Y. Oh H. (2016). The gender association of the SIRT1 rs7895833 polymorphism with pediatric obesity: a 3-year panel study. Lifestyle Genom. 9 (5–6), 265–75. 10.1159/000454713 28118644

[B52] Listenberger L. L. Han X. Lewis S. E. Cases S. Farese Jr R. V. Ory D. S. (2003). Triglyceride accumulation protects against fatty acid-induced lipotoxicity. Proc. Natl. Acad. Sci. 100 (6), 3077–3082. 10.1073/pnas.0630588100 12629214 PMC152249

[B53] Luan J. Browne P. O. Harding A.-H. Halsall D. J. O’Rahilly S. Chatterjee V. K. K. (2001). Evidence for gene-nutrient interaction at the PPARgamma locus. Diabetes 50 (3), 686–689. 10.2337/diabetes.50.3.686 11246892

[B54] MacDougald O. A. Hwang C.-S. Fan H. Lane M. D. (1995). Regulated expression of the obese gene product (leptin) in white adipose tissue and 3T3-L1 adipocytes. Proc. Natl. Acad. Sci. 92 (20), 9034–9037. 10.1073/pnas.92.20.9034 7568067 PMC40918

[B55] Masud S. Ye S. SAS Group (2003). Effect of the peroxisome proliferator activated receptor-γ gene Pro12Ala variant on body mass index: a meta-analysis. J. Med. Genet. 40 (10), 773–780. 10.1136/jmg.40.10.773 14569127 PMC1735275

[B56] Meneguette M. V. de O. Oliveira C. A. de Lima M. H. de M. Pina K. N. Amaral M. E. C. do (2016). Polymorphism in the SIRT1 gene and parameters of metabolic syndrome in a sample of the adult Brazilian population. Rev. Nutr. 29, 1–10. 10.1590/1678-98652016000100001

[B57] Mertens I. Verrijken A. Michiels J. J. Van der Planken M. Ruige J. B. Van Gaal L. F. (2006). Among inflammation and coagulation markers, PAI-1 is a true component of the metabolic syndrome. Int. J. Obes. 30 (8), 1308–1314. 10.1038/sj.ijo.0803189 16389265

[B58] Moreno-Navarrete J. M. Moreno M. Vidal M. Ortega F. Ricart W. Fernández-Real J. M. (2015). DBC1 is involved in adipocyte inflammation and is a possible marker of human adipose tissue senescence. Obesity 23 (3), 519–522. 10.1002/oby.20999 25682741

[B59] Nicoletti C. F. de Oliveira APRP Brochado M. J. F. Pinhel M. A. S. de Oliveira B. A. P. Marchini J. S. (2017). The Ala55Val and-866G> A polymorphisms of the UCP2 gene could be biomarkers for weight loss in patients who had Roux-en-Y gastric bypass. Nutrition 33, 326–330. 10.1016/j.nut.2016.07.020 27743836

[B60] Oberkofler H. Esterbauer H. Hell E. Krempler F. Patsch W. (2000). The Gln27Glu polymorphism in the beta2-adrenergic receptor gene is not associated with morbid obesity in Austrian women. Int. J. Obes. 24 (3), 388–390. 10.1038/sj.ijo.0801180 10757636

[B61] Obradovic M. Sudar-Milovanovic E. Soskic S. Essack M. Arya S. Stewart A. J. (2021). Leptin and obesity: role and clinical implication. Front. Endocrinol. (Lausanne) 12, 585887. 10.3389/fendo.2021.585887 34084149 PMC8167040

[B62] Oomen J. M. van Rossum C. T. M. Hoebee B. Saris W. H. M. van Baak M. A. (2005). beta2-adrenergic receptor polymorphisms and salbutamol-stimulated energy expenditure. J. Clin. Endocrinol. Metab. 90 (4), 2301–2307. 10.1210/jc.2004-1356 15687340

[B63] Pecqueur C. Alves-Guerra M.-C. Gelly C. Lévi-Meyrueis C. Couplan E. Collins S. (2001). Uncoupling protein 2, *in vivo* distribution, induction upon oxidative stress, and evidence for translational regulation. J. Biol. Chem. 276 (12), 8705–8712. 10.1074/jbc.M006938200 11098051

[B64] Picard F. Kurtev M. Chung N. Topark-Ngarm A. Senawong T. Machado de Oliveira R. (2004). Sirt1 promotes fat mobilization in white adipocytes by repressing PPAR-gamma. Nature 429 (6993), 771–776. 10.1038/nature02583 15175761 PMC2820247

[B65] Prakash J. Srivastava N. Awasthi S. Agarwal C. Natu S. Rajpal N. (2012). Association of PPAR‐γ gene polymorphisms with obesity and obesity‐associated phenotypes in north indian population. Am. J. Hum. Biol. 24 (4), 454–459. 10.1002/ajhb.22245 22410809

[B66] Qian L. Xu K. Xu X. Gu R. Liu X. Shan S. (2013). UCP2-866G/A, Ala55Val and UCP3-55C/T polymorphisms in association with obesity susceptibility—a meta-analysis study. PLoS One 8 (4), e58939. 10.1371/journal.pone.0058939 23560041 PMC3613358

[bib84] Ramkaran P. Khan S. Moodley D. Chuturgoon A. A. Phulukdaree A. (2016). Sirtuin 1 rs1467568 and rs7895833 in South African Indians with early-onset coronary artery disease. Cardiovasc. J. Afr. 27 (4), 213–7. 27841908 10.5830/CVJA-2015-085 PMC5340890

[B67] Reihsaus E. Innis M. MacIntyre N. Liggett S. B. (1993). Mutations in the gene encoding for the beta 2-adrenergic receptor in normal and asthmatic subjects. Am. J. Respir. Cell Mol. Biol. 8 (3), 334–339. 10.1165/ajrcmb/8.3.334 8383511

[bib85] Robitaille J. Després J. Perusse L. Vohl M. (2003). The PPAR‐gamma P12A polymorphism modulates the relationship between dietary fat intake and components of the metabolic syndrome: results from the Québec Family Study. Clin. Genet. 63 (2), 109–16. 12630956 10.1034/j.1399-0004.2003.00026.x

[B68] Rocha R. M. Barra G. B. Rosa ÉCCC Garcia É. C. Amato A. A. Azevedo M. F. (2015). Prevalence of the rs1801282 single nucleotide polymorphism of the PPARG gene in patients with metabolic syndrome. Arch. Endocrinol. Metab. 59, 297–302. 10.1590/2359-3997000000086 26331316

[B69] Saraswati M. R. Suastika K. Malik S. G. Sudoyo H. (2011). The uncoupling protein 2 Ala55val polymorphism is associated with diabetes mellitus in a Balinese population. J. ASEAN Fed. Endocr. Soc. 26 (1), 39–43. 10.15605/jafes.026.01.08

[B70] Sathishkumar C. Prabu P. Balakumar M. Lenin R. Prabhu D. Anjana R. M. (2016). Augmentation of histone deacetylase 3 (HDAC3) epigenetic signature at the interface of proinflammation and insulin resistance in patients with type 2 diabetes. Clin. Epigenetics 8 (1), 125–212. 10.1186/s13148-016-0293-3 27904654 PMC5122206

[B71] Shimoyama Y. Suzuki K. Hamajima N. Niwa T. (2011). Sirtuin 1 gene polymorphisms are associated with body fat and blood pressure in Japanese. Transl. Res. 157 (6), 339–347. 10.1016/j.trsl.2011.02.004 21575918

[bib86] Silva S. de S. Leite N. Furtado-Alle L. de Souza R. L. R. Corazza P. R. P. Tradiotto M. C. (2022). ADRB2 gene influences responsiveness to physical exercise programs: a longitudinal study applied to overweight or obese Brazilian children and adolescents. Gene. 820, 146296. 35149152 10.1016/j.gene.2022.146296

[B72] Souza B. M. de Assmann T. S. Kliemann L. M. Gross J. L. Canani L. H. Crispim D. (2011). The role of uncoupling protein 2 (UCP2) on the development of type 2 diabetes mellitus and its chronic complications. Arq. Bras. Endocrinol. Metabol. 55, 239–248. 10.1590/s0004-27302011000400001 21779625

[B73] Spiegelman B. M. (1998). PPAR-gamma: adipogenic regulator and thiazolidinedione receptor. Diabetes 47 (4), 507–514. 10.2337/diabetes.47.4.507 9568680

[B74] Tao T. T. Lin X. H. Tang S. J. Gui W. W. Zhu W. F. Li H. (2022). Association of genetic variants in the Sirt1 and Nrf2 genes with the risk of metabolic syndrome in a Chinese Han population. BMC Endocr. Disord. 22 (1), 84–88. 10.1186/s12902-022-00965-0 35365152 PMC8973505

[B75] Unger R. H. Scherer P. E. (2010). Gluttony, sloth and the metabolic syndrome: a roadmap to lipotoxicity. Trends Endocrinol. Metab. 21 (6), 345–352. 10.1016/j.tem.2010.01.009 20223680 PMC2880185

[B76] Wang M.-Y. Grayburn P. Chen S. Ravazzola M. Orci L. Unger R. H. (2008). Adipogenic capacity and the susceptibility to type 2 diabetes and metabolic syndrome. Proc. Natl. Acad. Sci. 105 (16), 6139–6144. 10.1073/pnas.0801981105 18413598 PMC2329698

[B77] Xie W. He R. Zhang J. He Y. Wan Z. Zhou C. (2019). β‑blockers inhibit the viability of breast cancer cells by regulating the ERK/COX‑2 signaling pathway and the drug response is affected by ADRB2 single‑nucleotide polymorphisms. Oncol. Rep. 41 (1), 341–350. 10.3892/or.2018.6830 30542705

[B78] Xiu L. L. Weng J. P. Sui Y. Wang J. Yan J. H. Huang Z. M. (2004). Common variants in beta 3-adrenergic-receptor and uncoupling protein-2 genes are associated with type 2 diabetes and obesity. Zhonghua Yi Xue Za Zhi 84 (5), 375–379. 15061987

[B79] Yu X. Jacobs Jr D. R. Schreiner P. J. Gross M. D. Steffes M. W. Fornage M. (2005). The uncoupling protein 2 Ala55Val polymorphism is associated with diabetes mellitus: the CARDIA study. Clin. Chem. 51 (8), 1451–1456. 10.1373/clinchem.2004.044859 15951317

[B80] Zhang H. Wu J. Yu L. (2014). Association of Gln27Glu and Arg16Gly polymorphisms in Beta2-adrenergic receptor gene with obesity susceptibility: a meta-analysis. PLoS One 9 (6), e100489. 10.1371/journal.pone.0100489 24960039 PMC4069060

[B81] Zhao X. Gang X. He G. Li Z. Lv Y. Han Q. (2020). Obesity increases the severity and mortality of influenza and COVID-19: a systematic review and meta-analysis. Front. Endocrinol. (Lausanne) 11, 595109. 10.3389/fendo.2020.595109 33408692 PMC7779975

[B82] Zillikens M. C. van Meurs J. B. J. Rivadeneira F. Amin N. Hofman A. Oostra B. A. (2009). SIRT1 genetic variation is related to BMI and risk of obesity. Diabetes 58 (12), 2828–2834. 10.2337/db09-0536 19741164 PMC2780870

