# Supplementary material for: Genetic variants in DBC1, SIRT1, UCP2 and ADRB2 as potential biomarkers for severe obesity and metabolic complications
Source: Front Genet. 2024 May 22;15:1363417. doi: 10.3389/fgene.2024.1363417 (PMC11151296; doi:10.3389/fgene.2024.1363417)
Supplement: Supplementary file 1 [file Table1.docx]

**Supplemental Table S1:** Information on probe sequence used in this study

| **Gene** | **Polymorphisms** | **Probe Sequence (5' - 3')** | **Probe status** |
| --- | --- | --- | --- |
| ***DBC1*** | rs17060940 | TTTTGTTTTGTATCCCTGATCTCTT**[C/T]**TTGCAGTTTTTGCTGGGCAGGAAAG | Functionally Tested |
| ***SIRT1*** | rs7895833 | TGAGGTGGTAAAAGGCCTACAGGAA**[A/G]**TCAACGTAATGGAGATTAGGAAGCA | Functionally Tested |
|  | rs1467568 | TCCTACTCTTTCACTTAAACCCCAA**[A/G]**TGGCCAAGCTAGGATTGATTTGGTG | Functionally Tested |
| ***UCP2*** | rs660339 | CATCACACCGCGGTACTGGGCGCTG**[A/G]**CTGTAGCGCGCACTGGCCCCTGACT | Validated |
| ***PPARG*** | rs1801282 | AACTCTGGGAGATTCTCCTATTGAC**[C/G]**CAGAAAGCGATTCCTTCACTGATAC | Validated |
| ***ADRB2*** | rs1042713 | CAGCGCCTTCTTGCTGGCACCCAAT**[A/G]**GAAGCCATGCGCCGGACCACGACGT | Functionally Tested |
|  | rs1042714 | TGCGCCGGACCACGACGTCACGCAG**[C/G]**AAAGGGACGAGGTGTGGGTGGTGGG | Functionally Tested |
