# Supplementary material for: Genetic variants in DBC1, SIRT1, UCP2 and ADRB2 as potential biomarkers for severe obesity and metabolic complications
Source: Front Genet. 2024 May 22;15:1363417. doi: 10.3389/fgene.2024.1363417 (PMC11151296; doi:10.3389/fgene.2024.1363417)
Supplement: Supplementary file 2 [file Table2.docx]

**Supplemental Table S2:** Information of Hardy-Weinberg equilibrium

| **Gene** | **dbSNP** | **HWE (control)** | |
| --- | --- | --- | --- |
|  |  | **χ^2^** | ***p*** |
| *DBC1* | rs17060940 | 2.975 | 0.084 |
| *SIRT1* | rs7895833 | 0.012 | 0.912 |
|  | rs1467568 | 1.279 | 0.258 |
| *PPARG* | rs1801282 | 0.698 | 0.403 |
| *UCP2* | rs660339 | 0.565 | 0.452 |
| *ADRB2* | rs1042713 | 4.372 | **0.036** |
|  | rs1042714 | 2.096 | 0.147 |
