# Supplementary material for: Genetic variants in DBC1, SIRT1, UCP2 and ADRB2 as potential biomarkers for severe obesity and metabolic complications
Source: Front Genet. 2024 May 22;15:1363417. doi: 10.3389/fgene.2024.1363417 (PMC11151296; doi:10.3389/fgene.2024.1363417)
Supplement: Supplementary file 3 [file Table3.docx]

**Supplemental Table S3:** Association of common variants studied and anthropometric, biochemical, and blood pressure parameters

| **Parameters** | ***DBC1* (rs17060940)** | | ***SIRT (rs7895833)*** | | ***SIRT (rs1467568)*** | | ***UCP2 (rs660339)*** | | ***PPARG (rs1801282)*** | | ***ADRB2 (rs1042714)*** | |
| --- | --- | --- | --- | --- | --- | --- | --- | --- | --- | --- | --- | --- |
|  | *Genotypes (n)* | *Values* | *Genotypes (n)* | *Values* | *Genotypes (n)* | *Values* | *Genotypes (n)* | *Values* | *Genotypes (n)* | *Values* | *Genotypes (n)* | *Values* |
| **Weight (kg)** | CC (375) | 101.8 (65.5; 131.5) | AA (281) | 100.7 (63.8; 129,05) | AA (158) | 114.6 (69.4; 137.0) | GG (142) | 101.3 (65.7; 128.1) | CC (433) | 102.4 (65.1; 131.2) | CC (240) | 104.6 (67.0; 130.0) |
|  | CT+TT (126) | 112.2 (70.1; 134.2) | AG (198) | 110.0 (71.0; 135.8) | AG (238) | 104.3 (66.7; 134.5) | GA (245) | 101.5 (64.8; 131.3) | CG + GG (68) | 115.2 (78.4; 137.2) | CG (203) | 106.0 (65.0; 134.3) |
|  |  |  | GG (22) | 105.7 (65.7; 128.0) | GG (105) | 81.0 (64.2; 118.3) | AA (114) | 117.1 (72.0; 140.1) |  |  | GG (58) | 106.1 (68.6; 137.5) |
| β |  | 0.011 |  | 0.018 |  | -0.024 |  | 0.019 |  | 0.050 |  | 0.016 |
| *p* |  | 0.484 |  | 0.126 |  | 0.015 |  | 0.047 |  | 0.013 |  | 0.107 |
| **BMI (kg/m2)** | CC (375) | 39.5 (23.1; 47.8) | AA (281) | 37.5 (23.1; 46.9) | AA (158) | 43.4 (23.9; 50.4) | GG (142) | 38.1 (22.8; 46.5) | CC (433) | 39.7 (23.2; 47.5) | CC (240) | 39.7 (23.3; 47.3) |
|  | CT+TT (126) | 41.8 (24.1; 134.2) | AG (198) | 41.7 (24.1; 50.0) | AG (238) | 39.7 (23.2; 49.5) | GA (245) | 38.7 (23.3; 48.7) | CG + GG (68) | 44.3 (24.9; 52.2) | CG (203) | 40.8 (23.1; 49.8) |
|  |  |  | GG (22) | 44.0 (23.6; 47.3) | GG (105) | 24.9 (22.9; 45.5) | AA (114) | 44.3 (24.6; 51.8) |  |  | GG (58) | 39.8 (23.9; 49.5) |
| β |  | 0.009 |  | 0.023 |  | -0.024 |  | 0.025 |  | 0.050 |  | 0.016 |
| *p* |  | 0.557 |  | 0.053 |  | 0.012 |  | **0.008** |  | 0.011 |  | 0.120 |
| **BAI** | CC (368) | 41.3 (27.9; 51.3) | AA (277) | 38.5 (27.7; 50.8) | AA (157) | 45.6 (29.1; 52.2) | GG (140) | 39.4 (27.4; 50.0) | CC (426) | 41.3 (27.9; 51.2) | CC (236) | 41.7 (28.6; 51.4) |
|  | CT+TT (125) | 45.4 (28.6; 52.2) | AG (194) | 45.0 (29.3; 52.2) | AG (232) | 41.6 (29.3; 51.5) | GA (241) | 41.2 (27.7; 51.3) | CG + GG (67) | 47.0 (22.9; 52.2) | CG (201) | 42.4 (27.9; 51.8) |
|  |  |  | GG (22) | 47.8 (27.5; 53.6) | GG (105) | 32.5 (25.7; 50.3) | AA (112) | 45.2 (31.8; 54.8) |  |  | GG (58) | 41.3 (28.3; 53.0) |
| β |  | -0.001 |  | -0.003 |  | 0.011 |  | <0.001 |  | 0.007 |  | -0.002 |
| *p* |  | 0.837 |  | 0.515 |  | **0.002** |  | 0.973 |  | 0.312 |  | 0.567 |
| **Waist circumference (cm)** | CC (373) | 119.0 (83.1; 139.0) | AA (280) | 113.5 (82.6; 138.0) | AA (158) | 126.5 (85.0; 142.0) | GG (141) | 114.0 (81.5; 138.5) | CC (430) | 119.0 (83.0; 138.0) | CC (239) | 120.0 (84.0; 138.0) |
|  | CT+TT (125) | 124.0 (85.0; 142.0) | AG (196) | 124.7 (85.1; 142.0) | AG (235) | 120.0 (84.0; 140.0) | GA (241) | 118.0 (82.0; 138.0) | CG + GG (68) | 129.5 (95.1; 145.4) | CG (201) | 120.0 (82.2; 142.0) |
|  |  |  | GG (22) | 126.7 (83.7; 138.0) | GG (105) | 94.0 (81.2; 131.0) | AA (114) | 126.0 (89.7; 142.1) |  |  | GG (58) | 120.5 (83.7; 139.9) |
| β |  | -0.003 |  | -0.003 |  | 0.005 |  | -0.004 |  | 0.005 |  | 0.003 |
| *p* |  | 0.419 |  | 0.291 |  | 0.032 |  | 0.136 |  | 0.015 |  | 0.996 |
| **Hip circumference (cm)** | CC (373) | 125.5 (99.0; 144.0) | AA (280) | 123.5 (99.0; 143.2) | AA (158) | 132.5 (100.0; 148.0) | GG (141) | 123.5 (99.0; 142.7) | CC (430) | 126.0 (99.0; 145.6) | CC (239) | 128.0 (99.0; 142.0) |
|  | CT+TT (125) | 132.0 (100.5; 148.5) | AG (196) | 132.0 (102.0; 147.4) | AG (235) | 126.5 (100.5; 147.0) | GA (241) | 126.5 (99.0; 143.0) | CG + GG (68) | 135.0 (107.0; 146.0) | CG (201) | 127.0 (99.7; 148.0) |
|  |  |  | GG (22) | 130.5 (96.4; 146.7) | GG (105) | 106.0 (98.2; 136.5) | AA (114) | 134.0 (104.9; 150.0) |  |  | GG (58) | 129.7 (100.0; 148.0) |
| β |  | 0.001 |  | -0.004 |  | 0.007 |  | -0.003 |  | 0.004 |  | 0.002 |
| *p* |  | 0.740 |  | 0.060 |  | **>0.001** |  | 0.077 |  | 0.322 |  | 0.995 |
| **WHR** | CC (373) | 0.91 (0.83; 0.98) | AA (280) | 0.91 (0.83; 0.97) | AA (158) | 0.93 (0.85; 0.98) | GG (141) | 0.89 (0.83; 0.97) | CC (430) | 0.91 (0.83; 0.98) | CC (239) | 0.91 (0.84; 0.98) |
|  | CT+TT (125) | 0.91 (0.84; 0.98) | AG (196) | 0.91 (0.84; 0.98) | AG (235) | 0.91 (0.83; 0.98) | GA (241) | 0.91 (0.83; 0.98) | CG + GG (68) | 0.94 (0.86; 0.99) | CG (201) | 0.91 (0.83; 103.0) |
|  |  |  | GG (22) | 0.93 (0.85; 0.97) | GG (105) | 0.88 (0.82; 0.97) | AA (114) | 0.92 (0.87; 0.98) |  |  | GG (58) | 0.90 (0.83; 0.97) |
| β |  | -0.004 |  | 0.001 |  | -0.001 |  | <0.001 |  | 0.002 |  | <0.001 |
| *p* |  | 0.257 |  | 0.691 |  | 0.562 |  | 0.892 |  | 0.756 |  | 0.934 |
| **Glucose (mg/dl)** | CC (288) | 93.0 (86.2; 103.0) | AA (219) | 94.0 (86.0; 102.0) | AA (121) | 92.0 (87.0; 102.5) | GG (107) | 92.0 (87.0; 105.0) | CC (335) | 93.0 (87.0; 103.0) | CC (180) | 92.5 (87.0; 104.0) |
|  | CT+TT (100) | 92.0 (87.0; 101.7) | AG (153) | 92.0 (87.5; 103.0) | AG (181) | 94.0 (88.0; 105.5) | GA (194) | 93.5 (86.0; 101.2) | CG + GG (53) | 96.0 (89.0; 106.0) | CG (162) | 94.0 (87.0; 103.0) |
|  |  |  | GG (16) | 90.0 (86.0; 106.5) | GG (86) | 91.0 (85.0; 99.2) | AA (87) | 93.0 (88.0; 103.0) |  |  | GG (43) | 90.0 (87.0; 97.2) |
| β |  | 0.007 |  | -0.002 |  | 0.001 |  | -0.008 |  | -0.006 |  | -0.004 |
| *p* |  | 0.467 |  | 0.723 |  | 0.902 |  | 0.157 |  | 0.582 |  | 0.482 |
| **Total cholesterol (mg/dl)** | CC (318) | 187.0 (161.0; 218.2) | AA (243) | 187.0 (162.0; 217.0) | AA (135) | 187.0 (159.0; 218.0) | GG (121) | 187.0 (161.0; 211.0) | CC (370) | 185.5 (160.7; 216.0) | CC (205) | 186.0 (157.0; 218.0) |
|  | CT+TT (109) | 187.0 (156.5; 215.0) | AG (165) | 185.0 (159.0; 216.0) | AG (201) | 189.0 (161.0; 220.0) | GA (210) | 182.0 (159.0; 215.0) | CG + GG (57) | 192.0 (159.5; 222.0) | CG (172) | 185.0 (161.0; 216.0) |
|  |  |  | GG (19) | 188.0 (157.0; 216.0) | GG (91) | 184.0 (160.0; 211.0) | AA (96) | 193.0 (167.7; 231.0) |  |  | GG (50) | 191.5 (166.2; 215.5) |
| β |  | -0.008 |  | -0.010 |  | <0.001 |  | 0.012 |  | 0.007 |  | 0.002 |
| *p* |  | 0.415 |  | 0.157 |  | 0.953 |  | 0.051 |  | 0.549 |  | 0.708 |
| **HDL cholesterol (mg/dl)** | CC (318) | 52.0 (44.0; 61.2) | AA (243) | 52.0 (44.0; 63.0) | AA (135) | 50.0 (44.0; 61.0) | GG (121) | 50.0 (43.0; 60.0) | CC (370) | 51.0 (43.0; 61.2) | CC (205) | 52.0 (43.5; 61.0) |
|  | CT+TT (109) | 48.0 (43.0; 59.5) | AG (165) | 49.0 (43.0; 57.0) | AG (201) | 51.0 (43.0; 60.5) | GA (210) | 51.5 (43.0; 61.0) | CG + GG (57) | 48.0 (41.0; 57.5) | CG (172) | 50.0 (43.0; 60.0) |
|  |  |  | GG (19) | 52.0 (48.0; 69.0) | GG (91) | 51.0 (45.0; 62.0) | AA (96) | 49.5 (43.0; 62.7) |  |  | GG (50) | 50.5 (42.0; 61.2) |
| β |  | -0.012 |  | -0.006 |  | -0.005 |  | 0.009 |  | -0.011 |  | -0.005 |
| *p* |  | 0.258 |  | 0.403 |  | 0.414 |  | 0.183 |  | 0.414 |  | 0.417 |
| **LDL cholesterol (mg/dl)** | CC (313) | 110.0 (92.0; 135.0) | AA (239) | 110.0 (92.0; 137.0) | AA (134) | 109.0 (90.0; 139.0) | GG (121) | 109.0 (92.0; 132.0) | CC (363) | 109.0 (90.0; 132.0) | CC (202) | 107.5 (89.5; 133.0) |
|  | CT+TT (107) | 111.0 (91.0; 135.0) | AG (162) | 109.5 (90.0; 133.0) | AG (195) | 112.0 (91.0; 137.0) | GA (207) | 109.0 (88.0; 132.0) | CG + GG (57) | 121.0 (97.5; 144.0) | CG (168) | 111.0 (93.0; 137.0) |
|  |  |  | GG (19) | 115.0 (83.0; 137.0) | GG (91) | 108.0 (96.0; 122.0) | AA (94) | 114.5 (95.7; 145.2) |  |  | GG (50) | 114.5 (96.0; 139.0) |
| β |  | -0.001 |  | -0.015 |  | 0.007 |  | 0.009 |  | 0.026 |  | 0.011 |
| *p* |  | 0.965 |  | 0.145 |  | 0.412 |  | 0.305 |  | 0.146 |  | 0.227 |
| **Triglycerides (mg/dl)** | CC (318) | 105.5 (73.0; 146.2) | AA (243) | 101.0 (74.0; 136.0) | AA (135) | 104.0 (72.0; 139.0) | GG (121) | 99.0 (74.0; 140.5) | CC (370) | 100.5 (71.0; 136.2) | CC (205) | 100.0 (70.0; 140.0) |
|  | CT+TT (109) | 100.0 (74.0; 128.5) | AG (165) | 110.0 (74.5; 153.5) | AG (201) | 108.0 (77.0; 162.0) | GA (210) | 100.0 (69.7; 139.2) | CG + GG (57) | 114.0 (81.0; 166.5) | CG (172) | 106.0 (75.2; 146.0) |
|  |  |  | GG (19) | 91.0 (62.0; 127.0) | GG (91) | 95.0 (69.0; 122.0) | AA (96) | 110.5 (84.2; 139.7) |  |  | GG (50) | 103.0 (79.5; 133.2) |
| β |  | -0.032 |  | -0.023 |  | -0.001 |  | 0.006 |  | 0.021 |  | <0.001 |
| *p* |  | 0.151 |  | 0.154 |  | 0.929 |  | 0.668 |  | 0.462 |  | 0.975 |
| **Glycated hemoglobin** | CC (254) | 5.30 (4.90; 5.80) | AA (198) | 5.40 (5.00; 5.90) | AA (102) | 5.25 (4.90; 6.00) | GG (103) | 5.40 (4.90; 5.80) | CC (303) | 5.30 (4.90; 5.90) | CC (164) | 5.35 (5.00; 6.00) |
|  | CT+TT (88) | 5.40 (5.10; 6.00) | AG (129) | 5.30 (4.80; 6.00) | AG (163) | 5.30 (4.90; 5.90) | GA (177) | 5.30 (4.90; 5.90) | CG + GG (39) | 5.30 (5.00; 6.00) | CG (147) | 5.30 (4.90; 5.80) |
|  |  |  | GG (15) | 5.10 (4.90; 5.50) | GG (77) | 5.4 (4.90; 5.70) | AA (62) | 5.40 (4.90; 6.00) |  |  | GG (31) | 5.60 (5.00; 5.60) |
| β |  | 0.032 |  | -0.017 |  | 0.011 |  | -0.009 |  | -0.004 |  | -0.008 |
| *p* |  | **0.005** |  | 0.046 |  | 0.108 |  | 0.230 |  | 0.790 |  | 0.324 |
| **CRP** | CC (251) | 0.40 (0.13; 1.04) | AA (196) | 120.0 (110.5; 131.0) | AA (102) | 0.53 (0.13; 1.20) | GG (101) | 0.43 (0.10; 1.00) | CC (302) | 0.41 (0.12; 1.05) | CC (163) | 0.45 (0.12; 1.32) |
|  | CT+TT (87) | 0.50 (0.11; 1.28) | AG (127) | 0.65 (0.18; 1.32) | AG (161) | 0.51 (0.15; 1.18) | GA (176) | 0.39 (0.12; 1.00) | CG + GG (36) | 0.69 (0.10; 1.27) | CG (144) | 0.47 (0.15; 0.99) |
|  |  |  | GG (15) | 0.28 (0.09; 1.28) | GG (75) | 0.21 (0.09; 0.66) | AA (61) | 0.72 (0.15; 2.14) |  |  | GG (31) | 0.23 (0.09; 0.76) |
| β |  | 0.068 |  | 0.019 |  | -0.014 |  | 0.039 |  | -0.065 |  | -0.037 |
| *p* |  | 0.240 |  | 0.663 |  | 0.693 |  | 0.284 |  | 0.424 |  | 0.337 |
| **SBP** | CC (272) | 122.0 (112.0; 134.0) | AA (193) | 120.0 (110.5; 131.0) | AA (112) | 124.0 (115.0; 134.7) | GG (103) | 120.0 (111.0; 132.0) | CC (311) | 121.0 (112.0; 133.0) | CC (174) | 121.0 (112.0; 134.2) |
|  | CT+TT (83) | 120.0 (110.0; 131.0) | AG (145) | 124.0 (110.0; 136.0) | AG (164) | 122.0 (110.0; 137.0) | GA (174) | 123.0 (112.0; 134.2) | CG + GG (44) | 127.0 (110.0; 139.0) | CG (141) | 121.0 (110.0; 133.0) |
|  |  |  | GG (17) | 124.0 (118.0; 135.5) | GG (79) | 120.0 (110.0; 127.0) | AA (78) | 122.0 (110.7; 131.5) |  |  | GG (40) | 124.5 (118.2; 134.5) |
| β |  | -0.022 |  | -0.003 |  | 0.003 |  | -0.015 |  | -0.007 |  | 0.006 |
| *p* |  | 0.025 |  | 0.719 |  | 0.612 |  | 0.010 |  | 0.570 |  | 0.349 |
| **DBP** | CC (272) | 80.0 (72.0; 90.0) | AA (193) | 80.0 (72.0; 89.5) | AA (112) | 80.0 (70.2; 89.0) | GG (103) | 80.0 (70.0; 90.0) | CC (311) | 80.0 (71.0; 89.0) | CC (174) | 80.0 (71.0; 89.2) |
|  | CT+TT (83) | 78.0 (69.0; 87.0) | AG (145) | 80.0 (70.0; 90.0) | AG (164) | 80.0 (72.0; 90.0) | GA (174) | 80.0 (71.0; 89.0) | CG + GG (44) | 83.0 (69.2; 96.0) | CG (141) | 80.0 (70.0; 89.5) |
|  |  |  | GG (17) | 77.0 (67.5; 78.0) | GG (79) | 78.0 (70.0; 85.0) | AA (78) | 80.0 (71.0; 89.0) |  |  | GG (40) | 50.0 (69.2; 90.0) |
| β |  | -0.026 |  | -0.019 |  | 0.002 |  | -0.005 |  | 0.008 |  | <0.001 |
| *p* |  | 0.054 |  | 0.057 |  | 0.808 |  | 0.503 |  | 0.634 |  | 0.982 |
| **Leptin** | CC (90) | 2,254.1 (774.1; 3,019.6) | AA (72) | 2,032.2 (792.4; 2,822.8) | AA (33) | 2,858.5 (1,665.7; 3,611.6) | GG (34) | 2,237.4 (1,191.1; 3,112.7) | CC (105) | 2,219.3 (1,069.0; 2,882.8) | CC (54) | 2,227.3 (1,264.1; 3,109.2) |
|  | CT+TT (30) | 2,408.1 (1,869.7; 3,502.6) | AG (43) | 2,656.2 (1,617.7; 3,395.5) | AG (62) | 2,352.3 (1,571.8; 3,082.8) | GA (60) | 2,284.1 (915.5; 2,893.5) | CG + GG (15) | 2,858.5 (1,658.7; 3,354.6) | CG (48) | 2,274.5 (875.7; 3,258.4) |
|  |  |  | GG (17) | 2,627 (1,332.5; 4,178.7) | GG (25) | 1,325.3(537.6; 2,483.5) | AA (26) | 2,386.8 (1,563.5; 3,506.2) |  |  | GG (18) | 2,592.7 (1,065.2; 2,825.3) |
| β |  | 0.087 |  | 0.036 |  | -0.043 |  | -0.032 |  | 0.057 |  | 0.038 |
| *p* |  | 0.225 |  | 0.487 |  | 0.334 |  | 0.455 |  | 0.540 |  | 0.360 |
| **MCP1** | CC (90) | 257.6 (162.5; 361.6) | AA (72) | 257.1 (154.4; 353.4) | AA (33) | 240.8 (154.2; 382.4) | GG (34) | 249.0 (201.2; 406.7) | CC (105) | 257.2 (172.6; 365.9) | CC (54) | 240.3 (151.8; 333.7) |
|  | CT+TT (30) | 241.9 (164.7; 382.1) | AG (43) | 248.6 (200.7; 401.1) | AG (62) | 256.7 (188.2; 371.9) | GA (60) | 249.6 (154.8; 313.5) | CG + GG (15) | 254.4 (93.9; 304.3) | CG (48) | 264.1 (170.3; 382.7) |
|  |  |  | GG (17) | 314.5 (161.0; 424.5) | GG (25) | 258.0 (127.6; 338.5) | AA (26) | 288.2 (194.2; 399.8) |  |  | GG (18) | 258.1 (214.6; 388.3) |
| β |  | -0.059 |  | 0.058 |  | -0.038 |  | 0.003 |  | -0.105 |  | 0.012 |
| *p* |  | 0.308 |  | 0.170 |  | 0.283 |  | 0.931 |  | 0.165 |  | 0.716 |
| **PAI1** | CC (90) | 26,244.7 (19,219.7; 32,997.6) | AA (72) | 25,066.0 (19,133.4; 31,640.4) | AA (33) | 24,968.0 (18,716.9; 31,677.9) | GG (34) | 25,941.9 (20,114.9; 29,631.7) | CC (105) | 26,257.8 (19,886.0; 31.613.3) | CC (54) | 26,559.8 (18,543.4; 32,997.6) |
|  | CT+TT (30) | 25,587.5 (20,930.7; 30,958.7) | AG (43) | 27,013.8 (20,197.4; 31,688.3) | AG (62) | 27,171.7 (20,780.3; 32,997.6) | GA (60) | 24,810.1 (19,341.7; 31,335.0) | CG + GG (15) | 23,536.6 (16,573.4; 34,083.5) | CG (48) | 26,418.6 (20,895.9; 33,479.5) |
|  |  |  | GG (17) | 26,884.8 (23,742.3; 40,271.8) | GG (25) | 22,832.5 (19,007.7; 32,353.8) | AA (26) | 30,505.0 (19,453.6; 39,584.2) |  |  | GG (18) | 23,701.3 (18,043.4; 30,234.7) |
| β |  | 0.038 |  | 0.017 |  | 0.013 |  | 0.009 |  | -0.153 |  | <0.001 |
| *p* |  | 0.533 |  | 0.697 |  | 0.729 |  | 0.812 |  | 0.051 |  | 0.990 |
| **Resistin** | CC (90) | 8,210.9 (6,161.5; 10,578.4) | AA (72) | 8,434.1 (6,060.9; 10,715.6) | AA (33) | 8,331.7 (6,779.5; 10,489.1) | GG (34) | 8,111.1 (5,373.9; 10.702.5) | CC (105) | 8,331.7 (5,904.3; 10,646.5) | CC (54) | 8,114.9 (5,634.3; 10,361.0) |
|  | CT+TT (30) | 9,269.5 (6,145.3; 11,163.2) | AG (43) | 8,618.1 (6,436.9; 10,617.5) | AG (62) | 8,434.1 (6,161.5; 10,738.7) | GA (60) | 8,083.8 (5,889.5; 9,926.9) | CG + GG (15) | 9,072.8 (6,438.9; 10,679.4) | CG (48) | 9,059.0 (6,678.8; 11.609.5) |
|  |  |  | GG (17) | 7,187.7 (5,250.0; 10,079.8) | GG (25) | 8,585.3 (4,670.0; 10,512.3) | AA (26) | 9,559.4 (7,758.5; 14,186.9) |  |  | GG (18) | 7,201.9 (5,868.3; 10,559.2) |
| β |  | 0.014 |  | -0.003 |  | -0.002 |  | 0.075 |  | -0.029 |  | 0.031 |
| *p* |  | 0.804 |  | 0.951 |  | 0.927 |  | 0.023 |  | 0.688 |  | 0.334 |
